# Supplementary material for: Host-diet-gut microbiome interactions influence human energy balance: a randomized clinical trial
Source: Nat Commun. 2023 May 31;14:3161. doi: 10.1038/s41467-023-38778-x (PMC10232526; doi:10.1038/s41467-023-38778-x)
Supplement: Supplementary file 1 — Supplementary Information [file 41467_2023_38778_MOESM1_ESM.pdf]

Supplementary Materials

Host-Diet-Gut Microbiome Interactions Influence Human Energy Balance: a Randomized Clinical Trial

Karen D. Corbin[1], Elvis A. Carnero[1], Blake Dirks[2,3], Daria Igudesman[1], Fanchao Yi[1], Andrew Marcus[2,5], Taylor L. Davis[2,3], Richard E. Pratley[1], Bruce E. Rittmann[3,4], Rosa Krajmalnik-Brown*[2,4], Steven R. Smith*[1]

[1]AdventHealth Translational Research Institute, Orlando, Florida

[2]Biodesign Center for Health through Microbiomes, Arizona State University, Tempe, AZ

[3]Biodesign Swette Center for Environmental Biotechnology, Arizona State University, Tempe, AZ

[4]School of Sustainable Engineering and the Built Environment, Arizona State University, Tempe, AZ

[5]Skyology Inc, San Francisco, CA

Supplementary Tables

Supplementary Table 1: Dietary Intake Over 8 Inpatient Days

Nutrient	Target	Western Diet	Microbiome Enhancer Diet
Energy Intake (kcal/8 days)	Equivalence	17,008 ± 683	16,909 ± 700
Carbohydrates (%)	47-52%	47.91 ± 0.02%	49.18 ± 0.06 %
Fat (%)	32-37%	35.19 ± 0.01%	34.43 ± 0.04%
Protein (%)	15-18%	16.29 ± 0.009%	17.27 ± 0.03%
Fiber (g/1000 kcal)	6-10g/1000 kcal vs. 23-30g/1000 kcal	6.4 ± 0.02	26.0 ± 0.06
Resistant Starch, Estimated (g/1000 kcal)	<2g/1000 kcal vs. >8g/1000 kcals	1.2 ± 0	10.3 ± 0

Intake was evaluated by comparing the nutritional composition data from our menu design software to the amount of the provided diet consumed by each participant over the 8 inpatient days when all 3 meals were consumed on our metabolic ward and no modifications to the diets were made for enteroendocrine or food intake testing. Macronutrient content was based on a calculation that uses estimated average energy per gram of carbohydrate, protein, and fat. The total percentage of macronutrients approximates 100%. As a comprehensive database of resistant starch content of foods does not exist, the contributions for resistant starch were estimated based on published amounts of resistant starch in the foods provided[1]. Data reported as mean ± s.e.m.

Supplementary Table 2: Whole Room Calorimetry Schedule

Clock Time: Start	Clock Time: End	Activity
0800	0900	Participant to enter Calorimeter. Instruct to sit on bed supported by 30° wedge, feet in bed quietly and awake (NO TV/phone) x 1 hour. **No Restroom.**
0900	0930	Serve Breakfast. Participant to sit at desk. Eat 100 % in 30 min. (On scheduled day, instruct participant to ingest SmartPill after breakfast)
0930	1230	Participant in bed quietly (TV/phone ok) x 3 hours. **No Restroom.**
1230	1245	Participant to get ready for exercise. Use restroom.
1245	1315	Participant to walk on treadmill for 30 minutes: 3 mph and 1% incline
1315	1330	Recovery time. May use restroom.
1330	1400	Desk time for 30 min
1400	1430	Serve Lunch. Participant to sit at desk. Eat 100% in 30 min.
1430	1600	Participant in bed quietly (TV/phone ok) x 1.5 hours.
1600	1615	Participant to get ready for exercise. Use restroom
1615	1645	Participant to walk on Treadmill x 30 minutes: 3 mph and 1% incline.
1645	1700	Recovery time. May use restroom.
1700	1800	Desk time for 1 hour
1800	1830	Serve Dinner. Participant to sit at desk. Eat 100 % in 30 min.
1830	2000	Participant in bed quietly (TV/phone ok) x 1.5 hours.
2000	2200	Free Time x 2 hours.
2200	0600	Participant to empty bladder and collect urine prior to lights out. Curtains must be closed.
0600	0700	Awaken participant. May empty bladder. Participant in bed quietly x 1 hour.
0700		Participant to empty bladder, end of urine collection. Participant to exit Calorimeter.

Supplementary Table 3: Differential Relative Abundance by Diet at the Phylum and Family Levels

	P Value	Q Value
**Phylum Level**		
Firmicutes	0.041322	0.7438
Actinobacteria	0.36716	0.921388
Proteobacteria	0.591775	0.921388
Bacteroidetes	0.79006	0.94554
Verrucomicrobia	0.834099	0.94554
Euryarchaeota	0.95604	0.968383
**Family Level**		
Propionibacteriaceae	0.00766	0.402172
Oscillospiraceae	0.004497	0.402172
Actinomycetaceae	0.036966	0.880546
Clostridiales_Family_XIII_Incertae_Sedis	0.092248	0.880546
Acidaminococcaceae	0.051117	0.880546
Bifidobacteriaceae	0.532114	0.969746
Micrococcaceae	0.733506	0.969746
Atopobiaceae	0.817563	0.969746
Coriobacteriaceae	0.725051	0.969746
Eggerthellaceae	0.368079	0.969746
Bacteroidaceae	0.617749	0.969746
Barnesiellaceae	0.679615	0.969746
Odoribacteraceae	0.120954	0.969746
Prevotellaceae	0.274396	0.969746
Rikenellaceae	0.844536	0.969746
Tannerellaceae	0.639694	0.969746
Bacillales_unclassified	0.5078	0.969746
Lactobacillaceae	0.327826	0.969746
Leuconostocaceae	0.622826	0.969746
Streptococcaceae	0.524649	0.969746
Catabacteriaceae	0.278053	0.969746
Christensenellaceae	0.643426	0.969746
Clostridiaceae	0.669668	0.969746
Clostridiales_unclassified	0.639535	0.969746
Eubacteriaceae	0.539811	0.969746
Lachnospiraceae	0.528458	0.969746
Peptostreptococcaceae	0.647885	0.969746
Ruminococcaceae	0.152769	0.969746
Erysipelotrichaceae	0.415012	0.969746
Firmicutes_unclassified	0.838296	0.969746
Veillonellaceae	0.292069	0.969746
Akkermansiaceae	0.83385	0.969746
Desulfovibrionaceae	0.887671	0.972355
Methanobacteriaceae	0.956145	0.984267
Enterobacteriaceae	0.943787	0.984267

Phylum and family level differential abundance by diet tested by Compound Poisson linear mixed model. P-values were corrected to produce Q values using the Benjamini-Hochberg method.

Supplementary figures with legends

Supplementary Fig. 1

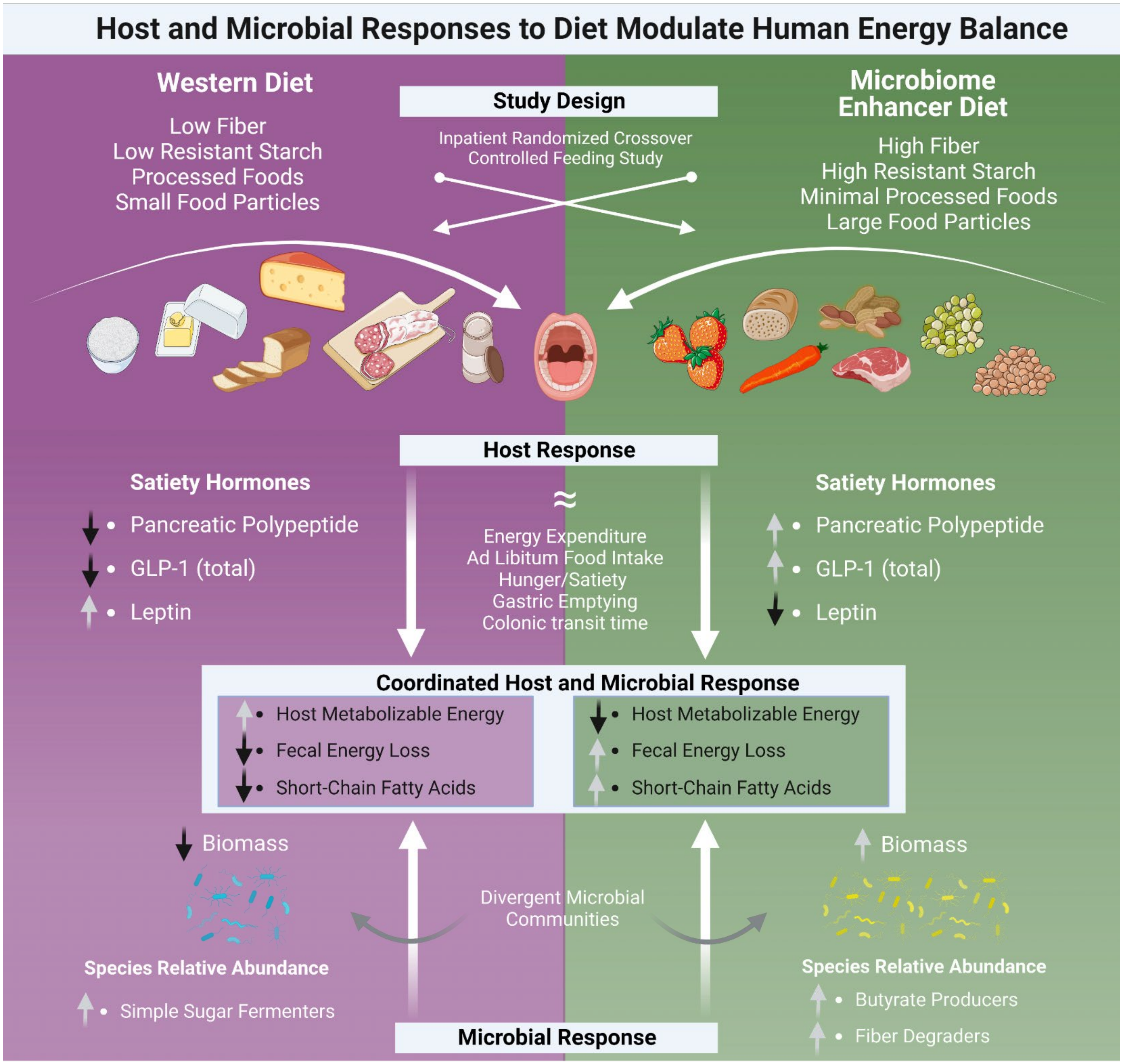

Supplementary Fig. 1. Study design and main findings. This schematic shows the key components of the study design, measurement endpoints, and summarizes main findings. Created with BioRender.com.

Supplementary Fig. 2

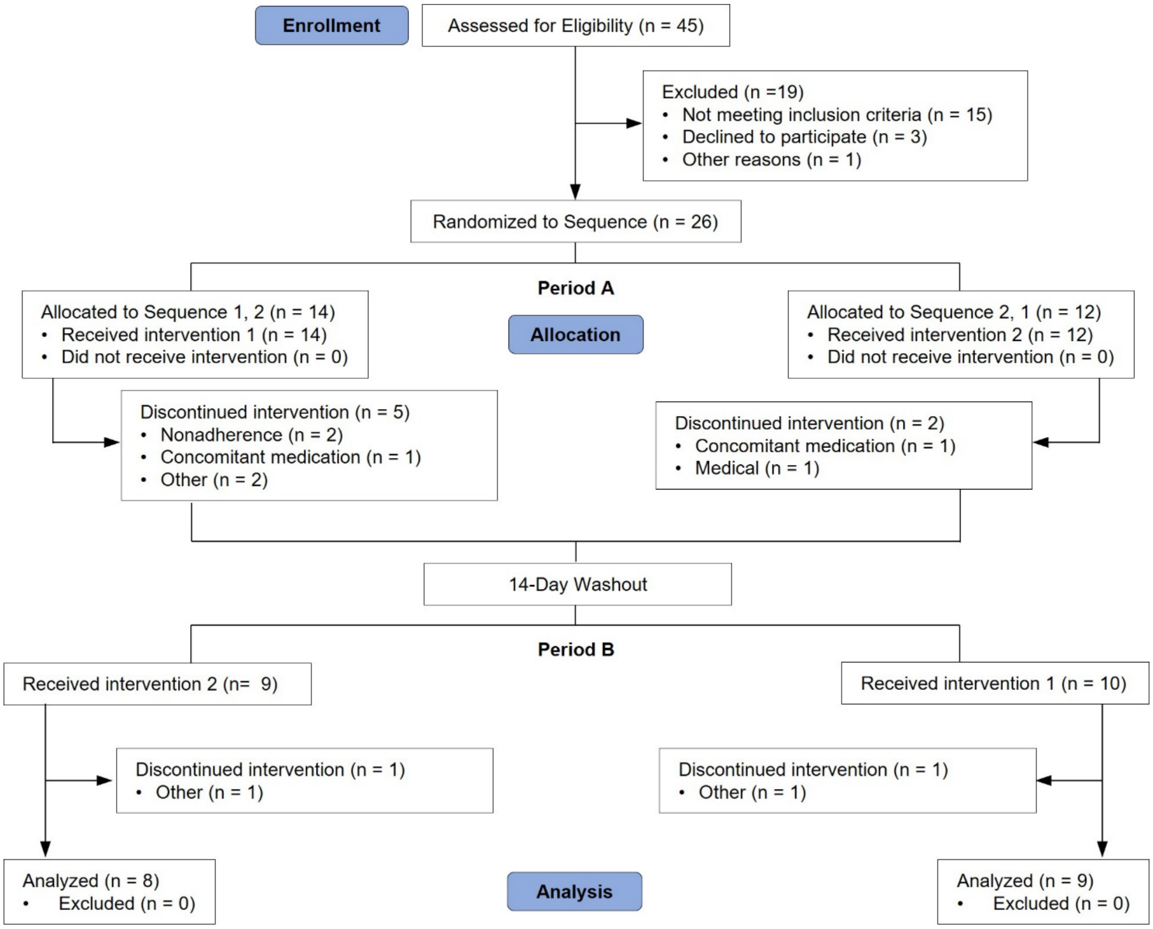

Supplementary Fig. 2 CONSORT diagram. This diagram shows the flow of participants from enrollment through analysis.

Supplementary Fig. 3

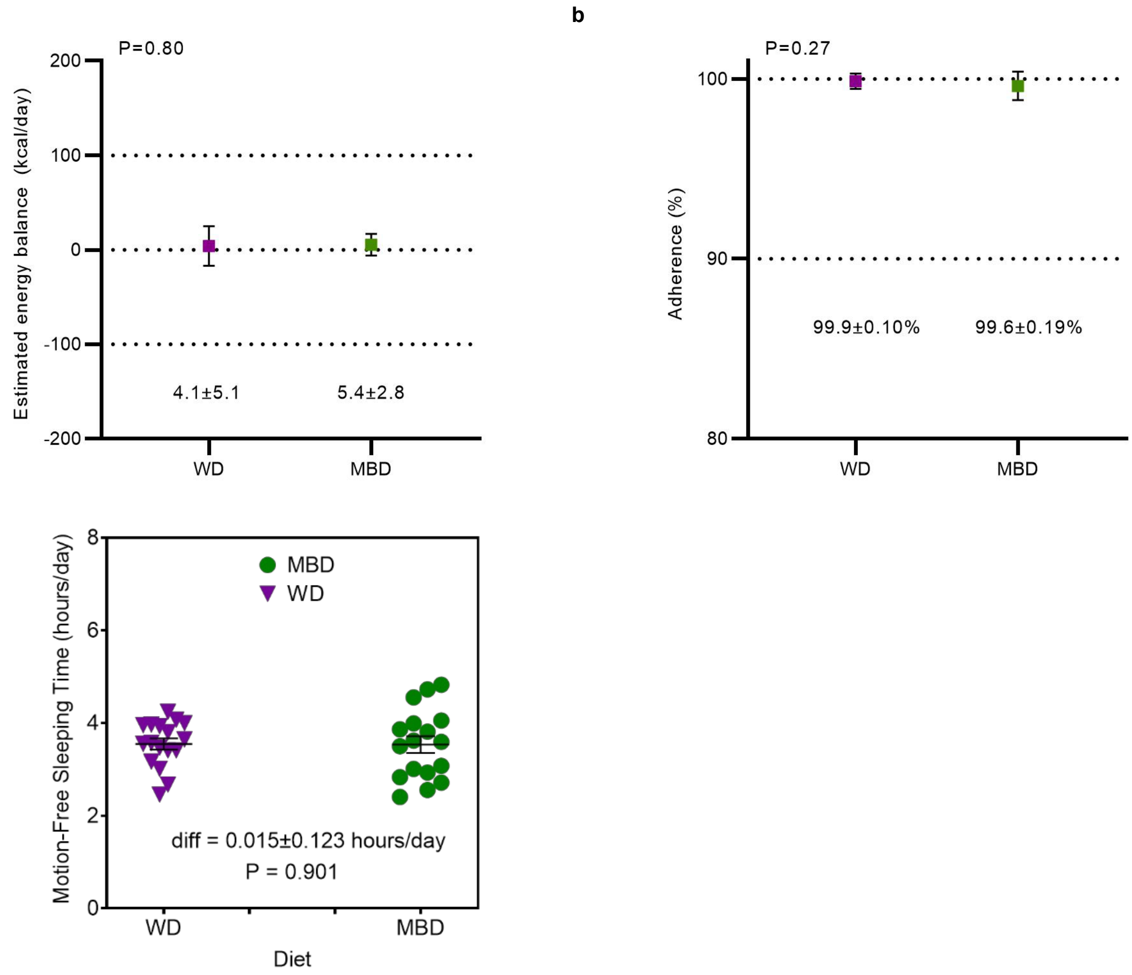

Supplementary Fig. 3. Experimental paradigm components: adherence, energy balance and sleep. a, Energy balance (mean of 6 measurement days) estimated from traditional parameters: Energy Balance = Energy Intake (kcals/24h) – Energy Expenditure (kcals/24h). **b,** Dietary adherence on the WD compared to the MBD over all inpatient days where all 3 meals were consumed on-site, and no changes were made to the feeding for testing. **c,** Motion-free sleeping time in hours (mean of 6 measurement days) calculated from the whole-room calorimeter radar sensor by removing all minutes with ≥ 6 counts of movement. N=17 per diet for both panels. Error bars in panels a and b are displayed as s.e.m. P values for panels a and b are from linear mixed effects regression models and denote the effect of diet (or lack thereof) on each endpoint. A paired sample t-test by diet was used to generate the P value in panel c. MBD—Microbiome Enhancer Diet (green); WD—Western Diet (purple).

Supplementary Fig. 4

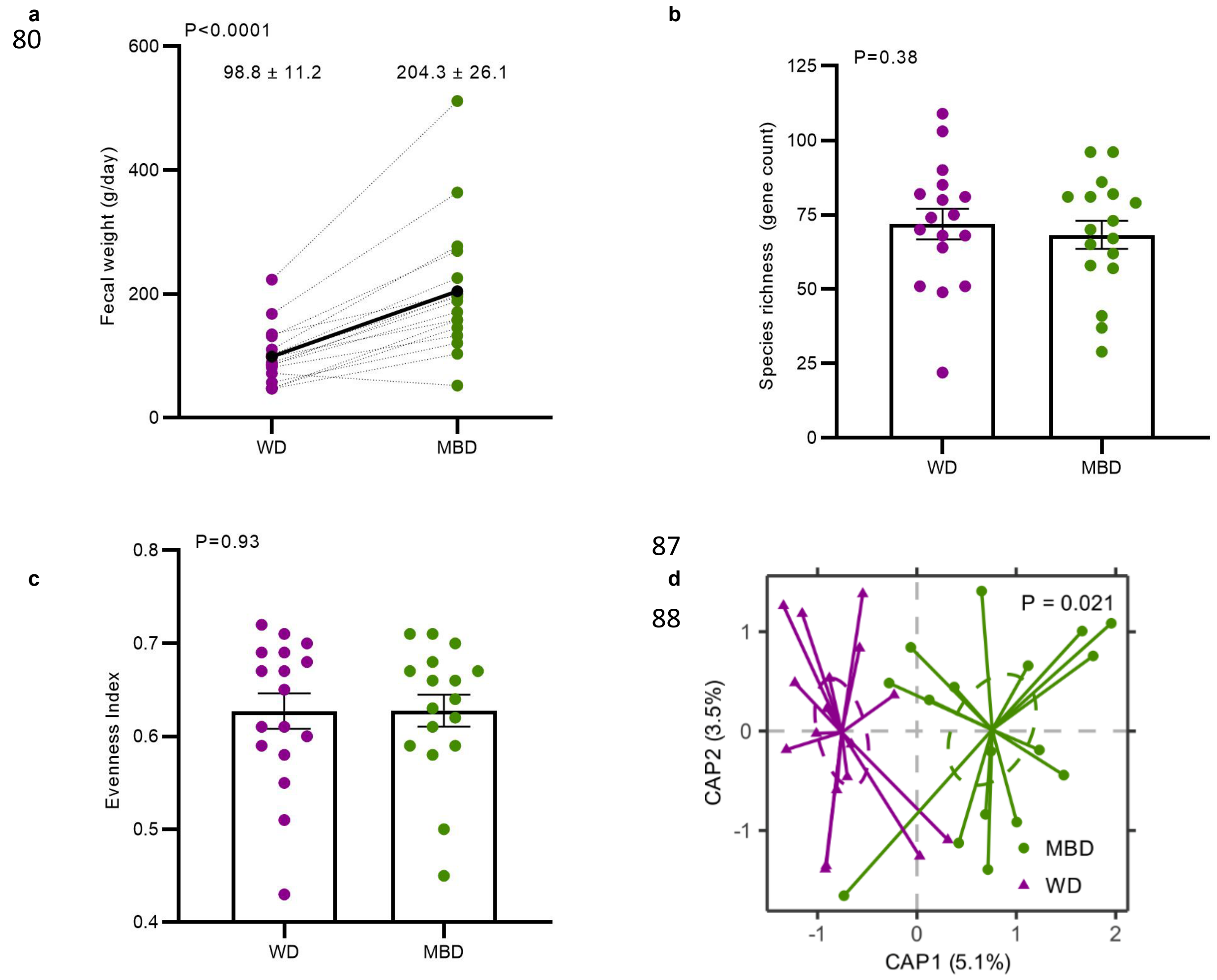

Supplementary Fig. 4. Gut microbiome community structure. a, Fecal weight from 6 days of composited feces averaged to generate daily production. Data reported as mean ± s.e.m. P = 1.24 x 10^{-5}. **b- c,** Alpha-diversity measures of richness and evenness. **d,** Beta-diversity at the species level assessed with Jaccard Similarity. Error bars in panels a and b are displayed as s.e.m. P values for panels a, b, and c are from linear mixed effects regression models and denote the effect of diet (or lack thereof) on each endpoint. P values for panel d are from permanova test and denote a statistically significant effect of diet on Jaccard Similarity metric. Confidence intervals for panel d are displayed as s.e.m. CAP—Canonical Analysis of Principal Coordinates; MBD—Microbiome Enhancer Diet (green); WD—Western Diet (purple).

Supplementary Fig. 5

a

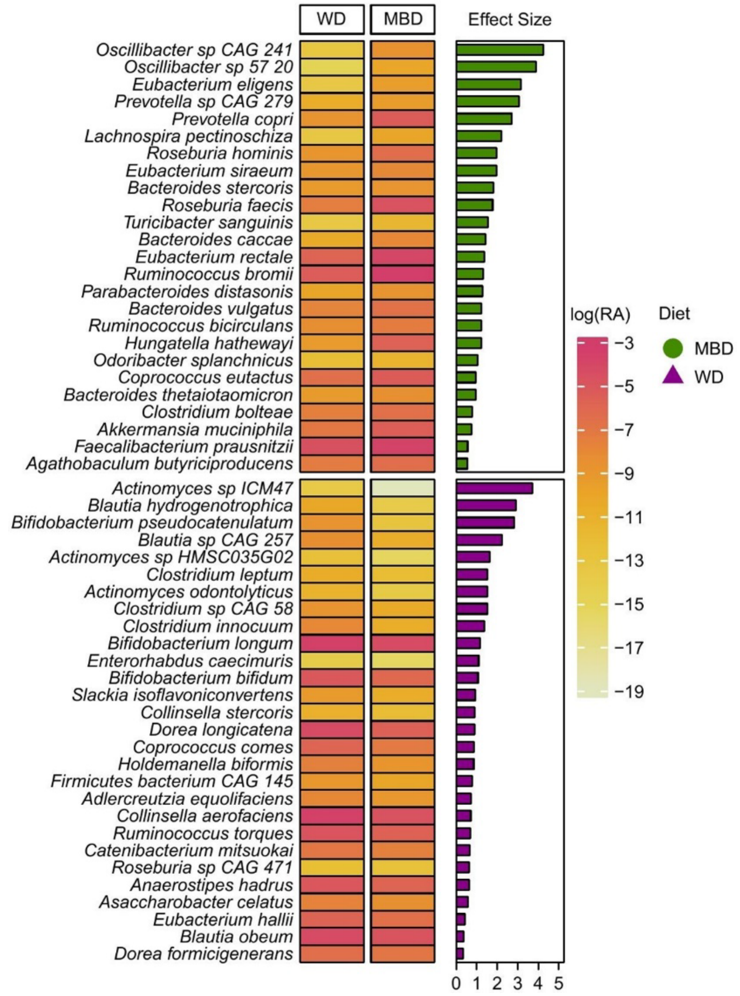

b

Species	Treatment	Q-value
Oscillibacter_sp_CAG_241	MBD	0.01
Oscillibacter_sp_57_20	MBD	2.27×10^{-07}
Eubacterium_eligens	MBD	7.44×10^{-07}
Prevotella_sp_CAG_279	MBD	0.0005
Prevotella_copri	MBD	1.46×10^{-06}
Lachnospira_pectinoschiza	MBD	0.001
Roseburia_hominis	MBD	0.002
Eubacterium_siraeum	MBD	0.07
Bacteroides_stercoris	MBD	0.04
Roseburia_faecis	MBD	0.001
Turicibacter_sanguinis	MBD	0.02
Bacteroides_caccae	MBD	0.02
Eubacterium_rectale	MBD	0.02
Ruminococcus_bromii	MBD	0.15
Parabacteroides_distasonis	MBD	0.03
Bacteroides_vulgatus	MBD	0.04
Ruminococcus_bicirculans	MBD	0.12
Hungatella_hathewayi	MBD	0.21
Odoribacter_splanchnicus	MBD	0.16
Coprococcus_eutactus	MBD	0.14
Bacteroides_thetaiotaomicron	MBD	0.22
Clostridium_bolteae	MBD	5.00×10^{-10}
Akkermansia_muciniphila	MBD	0.02
Faecalibacterium_prausnitzii	MBD	0.14
Agathobaculum_butyriciproducens	MBD	0.11
Dorea_formicigenerans	WD	0.10
Blautia_obeum	WD	0.14
Eubacterium_hallii	WD	0.14
Asaccharobacter_celatus	WD	$5.04E \times 10^{-06}$
Anaerostipes_hadrus	WD	4.37×10^{-05}
Roseburia_sp_CAG_471	WD	0.07
Catenibacterium_mitsuokai	WD	0.062
Ruminococcus_torques	WD	0.07
Collinsella_aerofaciens	WD	0.005
Adlercreutzia_equolifaciens	WD	5.68×10^{-09}
Firmicutes_bacterium_CAG_145	WD	0.0003
Holdemanella_biformis	WD	0.005
Coprococcus_comes	WD	5.68×10^{-09}
Dorea_longicatena	WD	1.26×10^{-05}
Collinsella_stercoris	WD	0.02
Slackia_isoflavoniconvertens	WD	0.002
Bifidobacterium_bifidum	WD	0.018
Enterorhabdus_caecimuris	WD	5.34×10^{-06}
Bifidobacterium_longum	WD	0.0003
Clostridium_innocuum	WD	0.07
Clostridium_sp_CAG_58	WD	0.07
Actinomyces_odontolyticus	WD	0.009
Clostridium_leptum	WD	0.02
Actinomyces_sp_HMSC035G02	WD	0.14
Blautia_sp_CAG_257	WD	0.001
Bifidobacterium_pseudocatenulatum	WD	5.60×10^{-05}
Blautia_hydrogenotrophica	WD	0.006
Actinomyces_sp_ICM47	WD	0.02

Supplementary Fig. 5. Gut microbiome remodeling in response to diet. a, Mean relative abundances and effect sizes of significantly differentially abundant species between diets. The heatmap shows the mean natural-log-transformed relative abundance (MaAsLin2) of species that were significantly different by diet. The bar graph shows the effect size of the regression coefficient from compound poisson regression models comparing species relative abundance by diet. Species shown in this figure had $P < 0.05$ and $Q < 0.25$. **b,** P-values were corrected to produce Q-values using the Benjamini-Hochberg method for each regression coefficient shown in **a**, which ranged from 5×10^{-10} to 0.217. The treatment indicates the diet on which the species relative abundance was higher. N=17 per diet for all panels. MBD—Microbiome Enhancer Diet; RA—relative abundance; WD—Western DietCAP—Canonical Analysis of Principal Coordinates; MBD—Microbiome Enhancer Diet (green); RA—relative abundance; WD—Western Diet (purple)

Supplementary Fig. 6

a

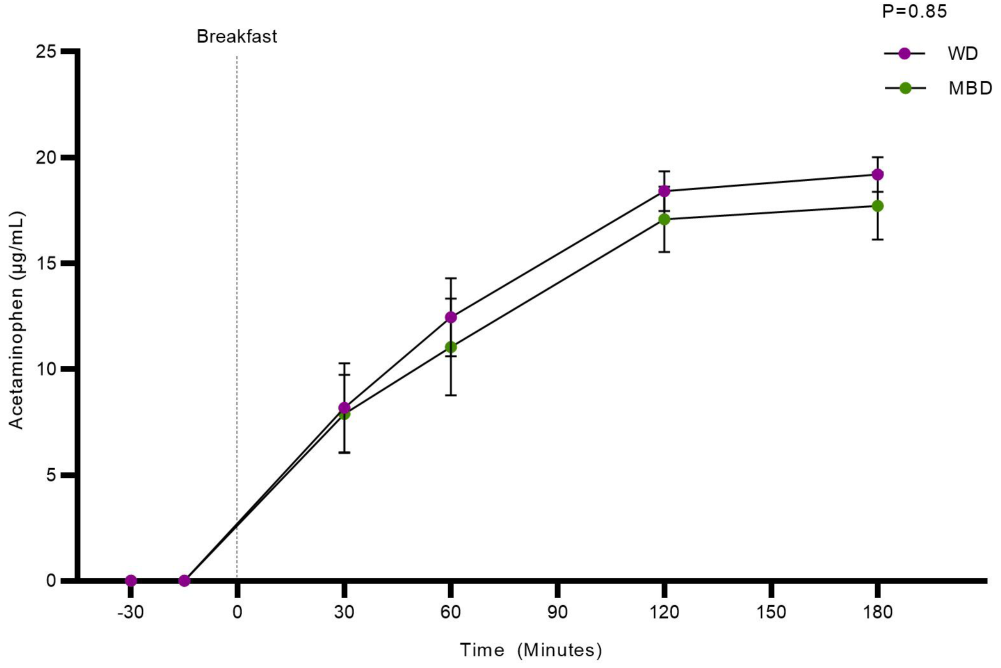

b

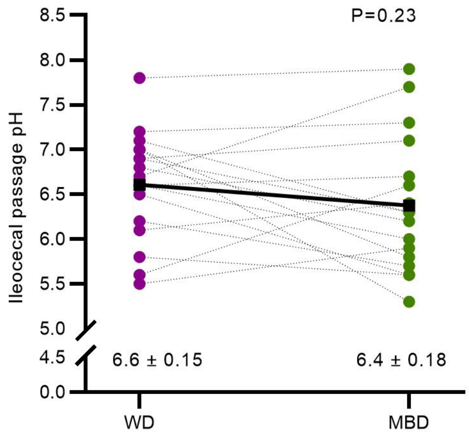

c

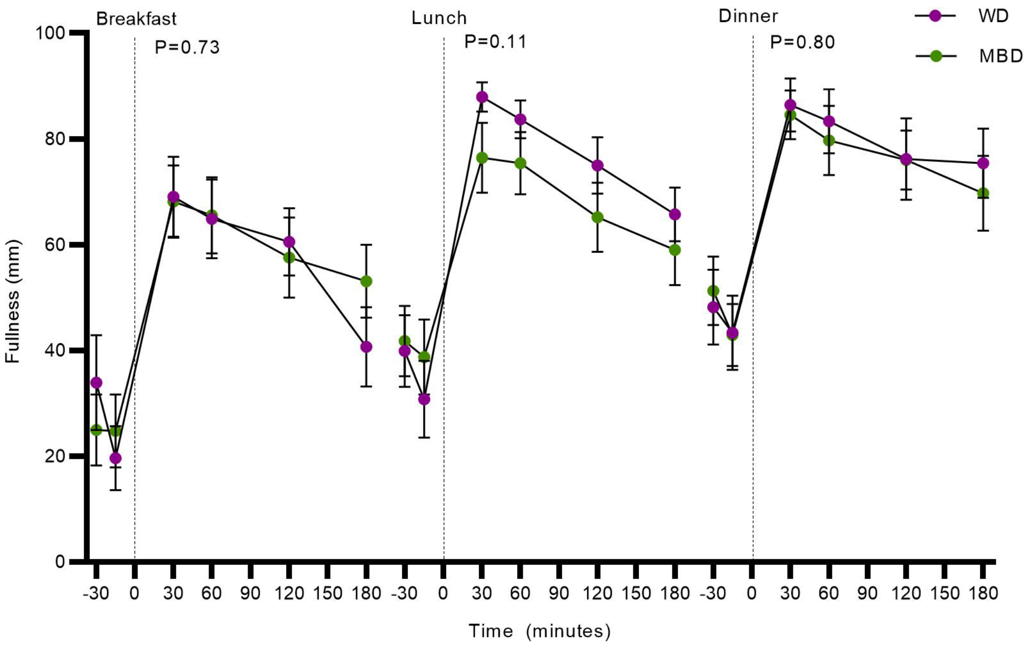
Breakfast
Lunch
Dinner
P=0.73
P=0.11
P=0.80
WD
MBD
Fullness (mm)
100
80
60
40
20
0
-30 0 30 60 90 120 150 180 -30 0 30 60 90 120 150 180 -30 0 30 60 90 120 150 180
Time (minutes)

d

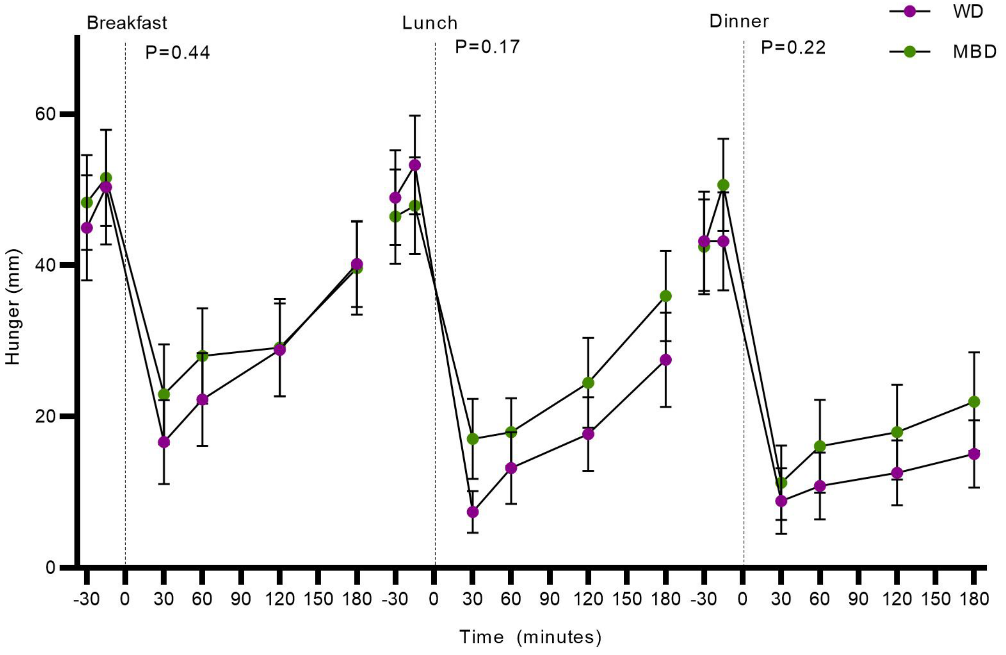
Breakfast
Lunch
Dinner
P=0.44
P=0.17
P=0.22
WD
MBD
Hunger (mm)
60
40
20
0
-30 0 30 60 90 120 150 180 -30 0 30 60 90 120 150 180 -30 0 30 60 90 120 150 180
Time (minutes)

e

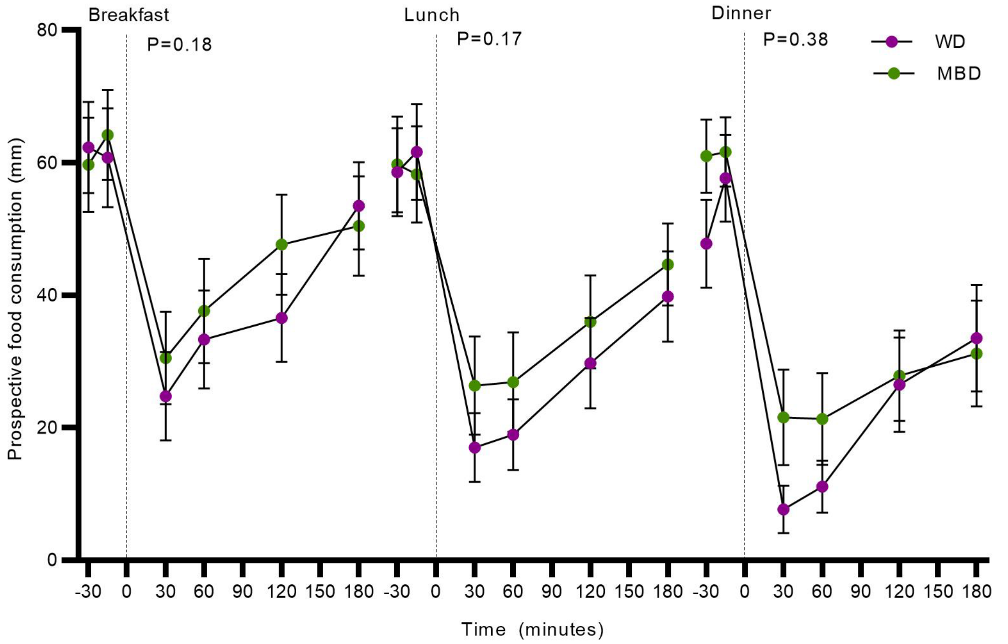

Breakfast
P=0.18
Lunch
P=0.17
Dinner
P=0.38
WD
MBD
Prospective food consumption (mm)
0
20
40
60
80
-30 0 30 60 90 120 150 180 -30 0 30 60 90 120 150 180 -30 0 30 60 90 120 150 180
Time (minutes)

f

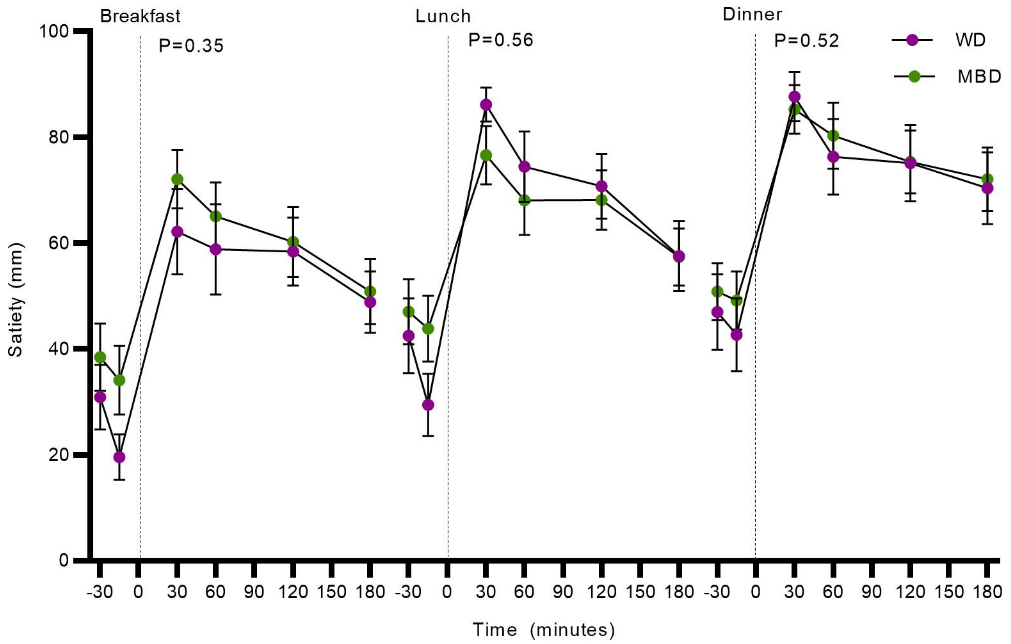

Breakfast
P=0.35
Lunch
P=0.56
Dinner
P=0.52
WD
MBD
Satiety (mm)
0
20
40
60
80
100
-30 0 30 60 90 120 150 180 -30 0 30 60 90 120 150 180 -30 0 30 60 90 120 150 180
Time (minutes)

g

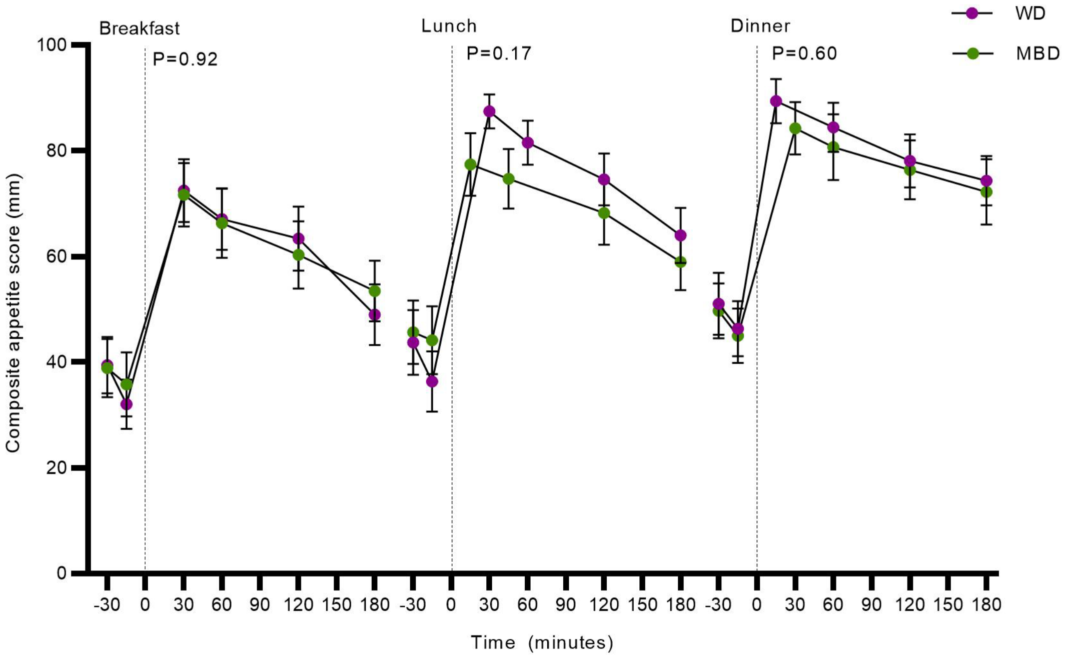

h

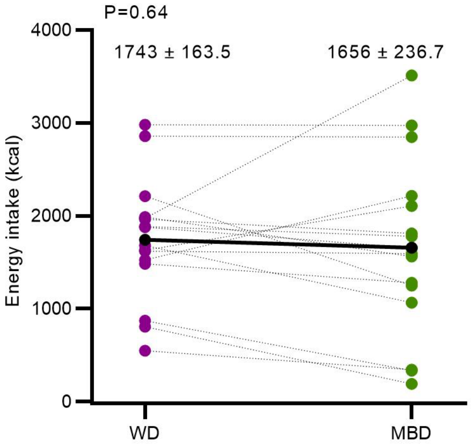

Supplementary Fig. 6. Host response to dietary intervention. a, Gastric emptying as evaluated by acetaminophen appearance after a fixed breakfast. **b,** pH within a 1-hour window of the ileocecal passage. **c-g,** Visual analog scale data for subjective ratings of fullness, hunger, prospective food consumption, satiety and a composite appetite score. **h,** Ad libitum energy intake evaluated during lunch and dinner after a fixed breakfast. All data reported as mean ± s.e.m. N=17 per diet for panels a and b; n=16 per diet for panels c-h. All error bars are displayed as s.e.m. P values for all panels are from linear mixed effects regression models and denote the effect of diet (or lack thereof) on each endpoint. MBD—Microbiome Enhancer Diet (green); WD—Western Diet (purple)

Supplementary Fig. 7

a

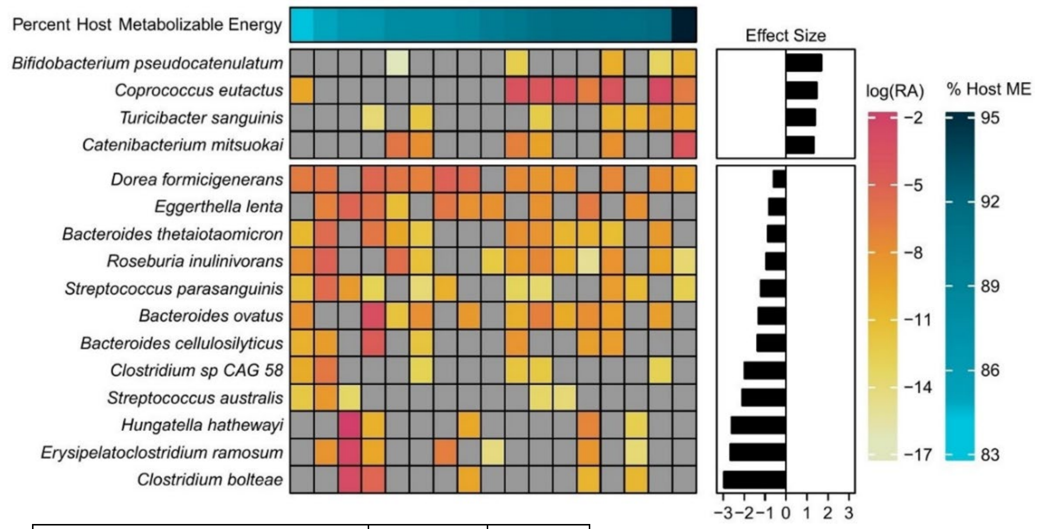

b

Species	Treatment	Q-value
Bifidobacterium_pseudocatenulatum	MBD	0.09
Coprococcus_eutactus	MBD	0.03
Turicibacter_sanguinis	MBD	0.08
Catenibacterium_mitsuokai	MBD	0.1
Dorea_formicigenerans	MBD	0.2
Eggerthella_lenta	MBD	0.2
Bacteroides_thetaiotaomicron	MBD	0.2
Roseburia_inulinivorans	MBD	0.2
Streptococcus_parasanguinis	MBD	0.2
Bacteroides_ovatus	MBD	0.2
Bacteroides_cellulosilyticus	MBD	0.2
Clostridium_sp_CAG_58	MBD	0.03
Streptococcus_australis	MBD	0.04
Hungatella_hathewayi	MBD	0.04
Erysipelatoclostridium_ramosum	MBD	0.03
Clostridium_bolteae	MBD	0.02

c

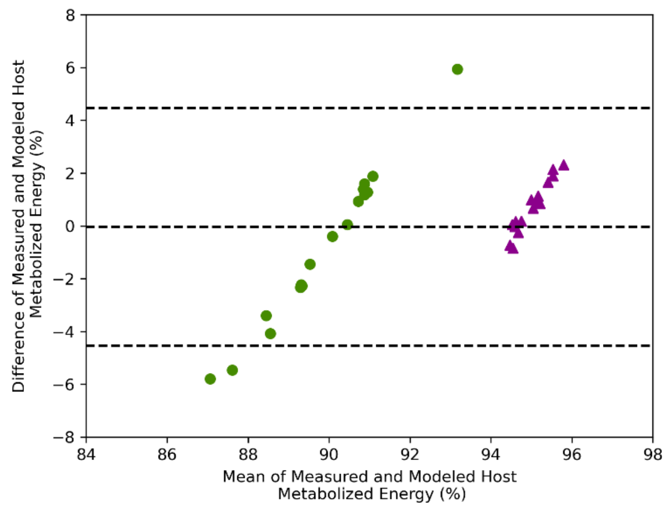

d

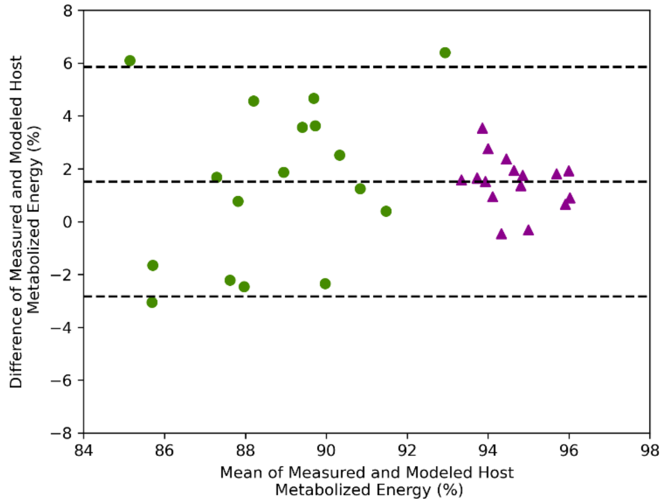

Supplementary Fig. 7 Microbial contributions to host energy balance. a, The heatmap shows the associations between host ME and mean natural-log-transformed relative abundance of each species (MaAsLin2). Each row is a species and each column is an individual participant. The bar graph shows the effect size of the regression coefficient between the independent variable of host metabolizable energy and each species, from compound Poisson regression models ($Q < 0.25$) **b,** P-values were correct to produce Q-values using the Benjamini-Hochberg method, for the regression coefficients shown in **a**, for each species (range 0.023 - 0.198). **c,** Bland-Altman plot comparing measured host metabolizable energy vs. modeled using a fixed CTT of 48 h. **d,** Bland-Altman plot comparing actual host metabolizable energy vs. modeled using the measured CTT for each participant. N=17 per diet for all panels. COD—chemical oxygen demand; Green—Western Diet; ME—metabolizable energy; Purple—Microbiome Enhancer Diet; RA—relative abundance

References

1 Murphy, M. M., Douglass, J. S. & Birkett, A. Resistant starch intakes in the United States. *Journal of the American Dietetic Association* **108**, 67-78, doi:10.1016/j.jada.2007.10.012 (2008).

2 Lewis, S. J. & Heaton, K. W. Stool form scale as a useful guide to intestinal transit time. *Scand J Gastroenterol* **32**, 920-924, doi:10.3109/00365529709011203 (1997).
