# Supplementary material for: Host-diet-gut microbiome interactions influence human energy balance: a randomized clinical trial
Source: Nat Commun. 2023 May 31;14:3161. doi: 10.1038/s41467-023-38778-x (PMC10232526; doi:10.1038/s41467-023-38778-x)
Supplement: Supplementary file 3 — Description of Additional Supplementary Files [file 41467_2023_38778_MOESM3_ESM.docx]

Description of Additional Supplementary Files

Supplementary Dataset 1: Adverse Events

Supplementary Dataset 2: ANCOM-BC Validation of Differential Abundance by Diet

Supplementary Dataset 3: Kendall’s tau-b correlation coefficient Validation of Correlations between Relative Abundance and Host Metabolizable Energy on the Microbiome Enhancer Diet

Supplementary Dataset 4: Study Protocol
