# Supplementary material for: Host-diet-gut microbiome interactions influence human energy balance: a randomized clinical trial
Source: Nat Commun. 2023 May 31;14:3161. doi: 10.1038/s41467-023-38778-x (PMC10232526; doi:10.1038/s41467-023-38778-x)
Supplement: Supplementary file 7 — Supplementary Dataset 4 [file 41467_2023_38778_MOESM7_ESM.pdf]

Study Protocol Title:
Integrating Quantitative Energetics Determines the Microbiome's Contribution to Energy Balance

Study Sponsor:
AdventHealth Orlando

Principal Investigator:
Steven R. Smith, MD
Chief Scientific Officer, AdventHealth Orlando Research
Senior Investigator, Translational Research Institute
301 E. Princeton St.
Orlando, FL 32804
407-303-7115

Sub-Investigators:
Translational Research Institute for Metabolism and Diabetes (TRI)
Richard Pratley, MD
Karen D. Corbin, PhD, RD
Heather Richmond, PA-C

Arizona State University (ASU)
Rosa Krajmalnik-Brown, PhD
Bruce Rittman, PhD
Andrew Marcus, PhD

Pennington Biomedical Research Center (PBRC)
Catherine Champagne, PhD, RD

Table of Contents:

Introduction 5
Background Information and Scientific Rationale 5
Study Objectives 6
Study Design 8
Overall Research Design 8
Study Agent, Device, and/or Intervention Description 10
Multi-Site Research Logistics/Communication Plan 10
Research Conducted in a Foreign Country 10
Community-Based Participatory Research 10
Study Site / Location(s) and Number of Participants 10
Participant Selection 11
Vulnerable Populations (if applicable) 11
Inclusion Criteria: 11
Exclusion Criteria: 11
Resources Available 13
Study Procedures 13
Participant Recruitment and Screening 13
Consent Process and Documentation 14
Randomization 14
Study Visits 14
Analytical / Clinical Procedures 21
Medical History and Physical Exam 21
Anthropometric Screening 21
Home Weight Checks: 21
Screening Labs 21
Vital Sign Measurements 21
Maintenance of energy balance in a whole-room calorimeter. 23
24 hour urine collection 23
24 hour fecal collection 23
Dual energy x-ray absorptiometry (DEXA) 23
Gastric Emptying 23
Diet History Questionnaire 24
Physical activity monitoring 24
Magnetic Resonance Imaging and Spectroscopy 24
Study Duration: 31

Materials of Human Origin: Collection, Preparation, Handling and Shipping 31
Study Outcomes Measures (Endpoints) 32
Data Management and Quality Plan 33
Data De-identification 33
Data Confidentiality, Storage, and Retention 33
Data Quality 34
Data Sharing (outside of AdventHealth Orlando) 34
Sample Size Determination 34
Power analysis: 34
Statistical Analysis Plan 35
Potential Risks and Benefits 36
Potential Benefits 36
Potential Risks 36
Mitigation of Risks 40
Provisions to Protect the Privacy Interest of Participants 40
Early Withdrawal of Participants 40
Investigator Withdrawal of Participants 40
Participant Request for Withdrawal from Study 40
Data Collection and Follow-up for Withdrawn Participants 41
Adverse Event Reporting 41
Adverse Events 41
Recording and Notification of Adverse Events 41
Safety Monitoring Plan 41
Safety Monitoring 41
Data and Safety Monitoring Board (DSMB) or Equivalent 41
Ethical Considerations 42
Sharing of Results with Participants 42
Funding Source 42
Participant Stipends or Payments 42
Publication Plan 43
References 43

List of Abbreviations:

BMI: Body Mass Index (kg/m^2)
BMP: Biochemical Methane Potential
BP: Blood Pressure
COD: Chemical Oxygen Demand
DEXA: Dual X-Ray Absorptiometry
EB: Energy Balance
ED: Emergency Department
EE: Energy Expenditure
EI: Energy Intake
FFQ: Food Frequency Questionnaire
ADVENTHEALTH ORLANDO: AdventHealth Orlando
GC: Gas Chromatograph or Gas Chromatography
ICP: Ileocecal passage
IHL: Intrahepatic Lipids
ME: Microbiome Enhancer
MFC: Mass Flow Controller
MRI/S: Magnetic Resonance Imaging and Spectroscopy
NIST: National Institute of Standards Technology
PBMC: Peripheral Blood Mononuclear Cell
PEG: Polyethylene Glycol
RMR: Resting Metabolic Rate
RQ: Respiratory Coefficient
SCFA: Short Chain Fatty Acids
SEE: Sleep Energy Expenditure
SOP: Standard Operating Procedure
TDEE: Total Daily Energy Expenditure
TEF: Thermic Effect of Feeding
VAS: Visual Analog Scale
VCH4: Volume of Methane Produced
VCO_2: Volume of Carbon Dioxide Produced
VO_2: Volume of Oxygen Consumed

Introduction

This document is a protocol for a human research study to investigate the role of the gut microbiota as a factor in obesity. The described study will be conducted in compliance with the protocol, Good Clinical Practices (GCP) International Conference on Harmonization (ICH) Guidelines (E6) for GCPs standards as adopted by the Food and Drug Administration (FDA) and associated Federal regulations, and all applicable institutional research requirements.

Important Notes: more detailed discussions of the science- Significance, Innovation, Approach, Rationale, including preliminary data- are covered in the attached NIH grant application.

Minor modifications have been made to clinical operations aspects of this protocol including adding a screening window, adding a window between accelerometry data collection and start of outpatient diet, re-numbering study days, correcting minor errors in grant (such as discrepant information in different sections of the grant), refining exclusion/inclusion criteria to clarify intent, addition of procedures (MRI/S) and changes in some of the collaborating individuals/labs. These are within the scope of the original grant and allowable by the NIH based on the discretion of the PI.

Background Information and Scientific Rationale

The environmental causes of the obesity epidemic are strongly debated. Animal models and early studies in humans shifted attention to the role of the gut microbiota as a factor in obesity.

Hypotheses relating the microbiota and body weight can be clustered into three general categories.

1) The composition of the colonic microbiota and the foods that support it alter the extraction of energy and the energy available for storage and/or oxidation by the host.

2) The gut microbiota affects energy intake and therefore body composition through changes in appetite and satiety.

3) Changes in the microbiota affect the host's energy expenditure via changes in bile-acid metabolism and/ or other mechanisms.

Clinical studies published to date have simply catalogued a dictionary of microbiome (microbiota genomic make-up) diversity and functions. Previous studies showed that the gut microbiome can be rapidly changed (at least temporarily) through diet interventions; however, these studies have not addressed in a systematic and quantitative way how these changes affect energy balance. What is missing is a quantitative approach that rigorously answers the question: "***Does the gut microbiota and its bioenergetics quantitatively change the absorption of nutrients, enteroendocrine secretions (affecting appetite, satiety, and therefore food intake), or energy expenditure?***"

This study will take an integrated approach to understand how the composition of a Western diet vs. Microbiome Enhancer diet play a pivotal role in the host's energy balance. With these two diets used as tools to modify the gut microbiota, we aim to understand how gut microbial ecology, microbial bioenergetics, and microbial metabolites influence energy balance and ultimately body weight: energy extraction, enteroendocrine secretions, and energy expenditure.

Note: The Microbiome Enhancer Diet is the result of the integration of a variety of approaches to maximally affect changes in the nutrients that the gut microbiome is exposed to and which are hypothesized to change the microbiome make-up and function. See **Table 1** for more details. All of these foods are normally consumed in the US and no food additives will be included.

Our study is the first of its kind to combine the state of the art and precise metabolic phenotyping capabilities of TRI-MD with advanced mathematical modeling and analytical tools to advance our understanding of how the microbiome contributes to human metabolic health.

Study Objectives

The **Primary Objective** of this project is to investigate the effects of diet-induced changes in gut microbiome and ultimately quantifiable energy balance of the host. This study will significantly improve our understanding of the fundamental biochemical and molecular factors linking how gut microbial ecology, microbial bioenergetics, and microbial metabolites influence energy balance and ultimately body weight: energy extraction, enteroendocrine secretions and energy expenditure.

A **Secondary Objective** is to provide fecal samples from deeply phenotyped volunteers to Arizona State University (ASU) scientists to study in their bench-top bioreactors. These samples and other biological samples may also be shared with other investigators in the future for studies of the impact of diet on fecal &/or microbial composition, and / or microbial composition on biological functions related to metabolism and energy balance. Examples include, but are not limited to other laboratory analyses or introduction of human feces into mice to assess the effects on key components of energy balance and ultimately the development of obesity.

Global Hypothesis
The gut microbiota contributes to the host's energy balance in a quantifiable way. We therefore can change the magnitude of that contribution by managing microbial interactions and microbial activity through manipulating the diet.

Specific Hypotheses for the clinical study
Hypothesis 1: Changing from a Western diet to a Microbiome Enhancer (ME) diet consisting of whole foods drastically alters the composition of the gut microbiome and its metabolic contributions to the host.

Hypothesis 2: These changes in the microbiome lead to measurable and meaningful changes in host energy balance via changes in i) energy absorption, ii), gut hormone secretion, and iii) the

host’s energy expenditure.

Project Aims

Aim 1 Create, test, and refine an integrated *in silico* model of energy balance in a metabolic ward setting using a typical Western diet vs. a Microbiome Enhancer (ME) diet.

<u>Overview</u> This study will take an integrated approach to understanding how the composition of the diet plays a pivotal role for the gut microbiome and, as a consequence, the host’s energy balance. We will develop, test, and refine an *in silico* model that integrates the human energy balance with the microbial ecology of the intestines. The goal is to quantify the microbial contribution to energy balance and how diet affects the balance.

Using bench scale bioreactors in the laboratory, we will collect critical microbiology parameters needed to refine and parameterize the model. These experiments will be conducted on fecal samples collected in the clinical study.

In the clinic, we will control the diet (e.g., dietary fiber and other key nutrients) and environmental conditions as we collect key clinical data (e.g., bowel transit time, microbial nucleic acids for sequencing, and methane production) and biological samples. The clinical data laboratory assessments (generated from collected biospecimens) will be inputs to develop, test, and refine the model of microbial ecology / metabolism. Once the model is well developed, we will compare model outputs [predictions] to directly measured [observed] energy absorption using state-of-the-art metabolic-ward techniques.

Aim 2 Explore the effect of a Western vs. ME diet on proximal and distal gut enteroendocrine secretions, gastric emptying, and bowel transit time and relate these results to subjective hunger/satiety and measured food intake.

<u>Overview</u> We will take advantage of the rigidly controlled metabolic ward studies to explore the effects of changes in diet and the subsequent changes in the microbial community on gut enteroendocrine secretions, gut function, stomach emptying, and bowel transit time. Then we will carefully measure food intake under rigidly controlled conditions. These parameters will then be used to expand and refine the *in silico* model of the long-term effects on body weight and composition.

Aim 3 Using modeled and measured energy balances, quantify the effect of a Western diet vs. ME diet on the microbial contribution to energy balance.

<u>Overview</u> We will use our integrated model to predict impacts on microbial bioenergetics (e.g., growth of microorganisms and their generation of short-chain fatty acids and methane) and human whole-body energy metabolism. In the clinic, we will directly measure (in a whole-room calorimeter) human + microbial energy metabolism (VO2, VCO2, and VCH4). Through this aim, we will be able to quantify the microbial contribution to energy balance and, most importantly, evaluate the feasibility of changing the microbial contribution by changing the diet.

Impact and future directions: These studies will, for the first time, quantify the microbial community's contributions to the host's energy balance. By integrating clinical measurements, bioreactor experiments, and mathematical modeling, we will be able to describe cause-and-effect mechanisms. The studies will allow us to distinguish among effects stemming from a net change in energy absorption vs. alterations in energy expenditure and effects on hunger/ satiety. Our innovative methods also will enable future studies on the interacting roles of diet, the gut microbiome, and human physiology and metabolism.

Study Design

Overall Research Design

This is a randomized (to diet) within participant, cross-over clinical study which combines outpatient feeding and inpatient metabolic ward testing and uses a multi-disciplinary integrative quantitative systems biology approach. The overall study duration is approximately 90 days: a 28-day screening period (inclusive of wait time), a 3-day period to collect data that will determine energy requirements, and two dietary intervention periods: the first is 32 days and the second is 23 days. **Figure 1** represents the overall study design.

Figure 1 Overview of the study

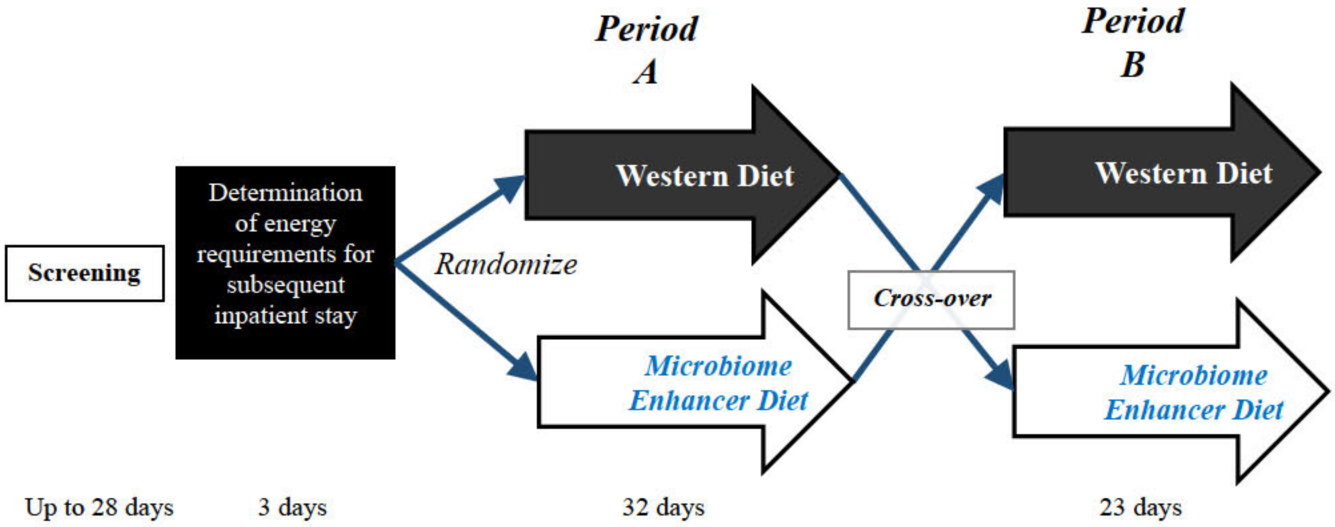

Overall Design for Each Project Aim

Aim 1 Create, test, and refine an integrated in silico model of energy balance in a metabolic ward setting using a typical Western diet vs. a Microbiome Enhancer (ME) diet.

A screening medical history, physical exam, anthropometric assessment, and lab work will exclude significant illness. Detailed Inclusion / Exclusion Criteria are listed elsewhere in the protocol.

After enrollment, participants will have free-living energy expenditure measured by armband and wrist accelerometry. Next, on Day 10 participants will consume a defined diet (Western vs. ME diet) starting and also start consuming PEG 0.5g TID to equilibrate the non-absorbable intake marker). On Day 11 they will be admitted for two nights and one full day in the calorimeter to assess energy requirements in the CRU. On Day 21 they will be admitted to the CRU and acclimatize for 3 days (21, 22, 23) on a diet designed to approximate energy balance. Days 24 – 29 will be spent in the calorimeter (23h in the calorimeter and 1 hour out to bathe). All urine and fecal matter will be collected for these 6 days. A second study period will start with Day 39. Full descriptions of all study days are found later in this protocol.

~~~~~~~~~~~~~~~~~~~~~~~~~~~~~~~~~~~~

Aim 2

Quantify the effect of a Western vs. ME diet on proximal and distal gut enteroendocrine secretions, gastric emptying, and bowel transit time and relate these results to subjective hunger/satiety and measured food intake. We will utilize the rigidly controlled metabolic-ward studies (see figure 2, above for context) to explore the effects of changes in diet and the subsequent changes in the microbial community on gut enteroendocrine secretions, gut function, gut emptying, and small-bowel transit time and food intake (Days 31-32).

In brief, on Day 30 the assigned diet will be continued and detailed enteroendocrine hormone profiles and gastric emptying assessments (acetaminophen concentrations) will be obtained from an indwelling IV catheter. On Day 31 ad libitum food intake will be measured and on the morning of Day 32 volunteers will be discharged from the CRU. A second study period will start with Day 39 +/- 7 with the second randomized diet. Full descriptions of all study days are found later in this protocol.

~~~~~~~~~~~~~~~~~~~~~~~~~~~~~

Aim 3

Using modeled and measured energy balances, quantify the effect of a Western diet vs. ME diet on the microbial contribution to energy balance. We will use our integrated model to predict diet impacts on microbial bioenergetics (e.g., growth of microorganisms and their generation of SCFA and methane) and human whole-body energy metabolism. In the clinic, we will directly measure (in a whole-room calorimeter) human + microbes energy metabolism. In Aim 3, we will quantify the microbial contribution to energy balance and, most importantly, evaluate the feasibility of changing the microbial contribution by changing the diet. This modeling work
~~~~~~~~~~~~~~~~~~~~~~~~~~~~~

occurs after the conclusion of the clinical study and is described in detail in the grant. No additional details for Aim 3 are provided in this protocol.

Study Agent, Device, and/or Intervention Description

N/A

Multi-Site Research Logistics/Communication Plan

AdventHealth Orlando TRI-MD is the single clinical site.

AdventHealth Orlando is sharing samples and data with other investigators.
- All other sites conducting work on samples collected during the clinical study will have the most current version of the protocol, consent document, and HIPAA authorization.
- All required approvals will have been obtained at each site (including approval by the site's IRB of record).
- All modifications will have been communicated to sites, and approved (including approval by the site's IRB of record) before the modification is implemented.
- All engaged participating sites will safeguard data as required by local information security policies.
- All local site investigators will conduct the study appropriately.
- Any non-compliance with the study protocol or applicable requirements will be reported in accordance with local policy.

Describe the method for communicating to participating sites:
- Problems - will be handled as per the attached MPI plan as submitted to NIH
- Interim results – N/A
- The closure of a study - will be handled as per the attached MPI plan as submitted to NIH

Research Conducted in a Foreign Country

N/A

Community-Based Participatory Research

N/A

Study Site / Location(s) and Number of Participants

The clinical aspects of the study will be conducted by the Investigators at the AdventHealth Orlando Translational Research Institute for Metabolism and Diabetes (TRI-MD). Approximately 18 participants will be enrolled during this study. The study will consist of all ethnic categories in proportions similar to the overall makeup of our community. We aim to enroll roughly equal numbers of men and women.

Participant Selection

Vulnerable Populations (if applicable)
AdventHealth Orlando Employees: Recruitment efforts will follow ADVENTHEALTH ORLANDO recruitment Standard Operating Procedures (SOPs) for research. ADVENTHEALTH ORLANDO Employees will not be individually targeted nor excluded from study participation based on employment. ADVENTHEALTH ORLANDO employees who engage the TRI-MD asking to participate in the study will be processed per standard consent procedures for participants. In addition, during the consent process, the study staff will review standard consent language stating that and employee's participation or lack of participation in the study will not affect their employment status or relationship with AdventHealth Orlando.

Participants enrolled in the study will be required to meet the following Inclusion / Exclusion criteria:

Inclusion Criteria:

1. Able to communicate meaningfully with the investigator and legally competent to provide informed written consent
2. Age 18 – 45 years, inclusive
3. Weight stable (+/- 3 kg) during the 6 months prior to enrollment
4. BMI ≤ 30 kg/m2

Exclusion Criteria:

Acute or chronic medical conditions or medication that would contraindicate the participation in the research testing or could potentially affect metabolic function including, but not limited to:

1. History or presence of cardiovascular disease (unstable angina, myocardial infarction or coronary revascularization within 6 months, presence of cardiac pacemaker, implanted cardiac defibrillator)
2. History of type 1 or type 2 diabetes
3. Bleeding disorders
4. Acute or chronic infections
5. Hepatitis and/or cirrhosis
6. Severe asthma or chronic obstructive pulmonary disease
7. Renal insufficiency or nephritis
8. Thyroid dysfunction (suppressed TSH, elevated TSH <10 µIU/ml if symptomatic or elevated TSH >10 µIU/ml if asymptomatic)
9. Uncontrolled hypertension (BP >160 mmHg systolic or >100 mmHg diastolic)
10. Prior bariatric surgery
11. Gastrointestinal disorders including: inflammatory bowel disease or malabsorption, swallowing disorders, suspected or known strictures, fistulas or physiological/mechanical GI obstruction, history of gastrointestinal surgery, Crohn's disease or diverticulitis.
12. Participants with strict dietary concerns (e.g. vegetarian or kosher diet, multiple food allergies, or allergies to food we will provide them during the study)
13. Current use of polyethylene glycol (e.g. Dulcolax, Miralax, Gavilax)

14. Cancer within the last 3 years (except non-melanoma skin cancer or treated cervical carcinoma in situ).
15. History of depression within < 5 years from screening visit or which, in the opinion of a medical investigator, will impact the participant's ability to complete the study.
16. History of eating disorders
17. Cushing's disease or syndrome
18. Untreated or inadequately controlled hypo- or hyperthyroidism
19. Active rheumatoid arthritis or other inflammatory rheumatic disorder
20. Pregnant or nursing females or females less than 6 months postpartum from the scheduled date of collection.
21. Tobacco use within the past 3 months
22. Metal implants (pace-maker, aneurysm clips) based on Investigator's judgment at Screening.
23. Unable to participate in MRI or MRS assessments due to physical limitations of equipment tolerances (e.g. MRI bore size) based on Investigator's judgment at Screening.
24. Unable to tolerate MRI/MRS imaging or claustrophobia.
25. Nickel allergy.
26. Had major surgery, donated or lost 1 unit of blood (approximately 500 mL) within 4 weeks prior to the pretrial (screening) visit.
27. Intolerance to acetaminophen use.
28. History of regular alcohol consumption exceeding 7 drinks/week for female participants or 14 drinks/week for male participants (1 drink = 5 ounces [150 mL] of wine or12 ounces [360 mL] of beer or 1.5 ounces [45 mL] of hard liquor) within 6 months before screening.
29. Anemia (hemoglobin <12 g/dl in men, <11 g/dl in women)

Excluded medications include, but are not limited to:
1. Nitrates
2. Anti-diabetic agents
3. Oral, injected or chronic topical steroids (inhaled steroids for mild asthma are acceptable)
4. Chronic use of aspirin or other non-steroidal anti-inflammatory drugs, including COX-2 inhibitors (a single aspirin daily if prescribed for cardioprotection will be allowed as will occasional use of aspirin and other non-steroidal drugs, provided that they are used for < 3 consecutive days and not during the period of metabolic testing)
5. Antibiotics taken in the last three months.
6. Use of any medications known to influence glucose, fat and/or energy metabolism within the last 3 months (e.g., OTC vitamins and supplements, growth hormone therapy, glucocorticoids [steroids], prescribed medications for weight loss, etc.)
7. Tricyclic antidepressants, atypical antipsychotics or other psychiatric drugs with effects on body weight

Study Restrictions:

Alcohol, Caffeine, Tobacco, and Activity

- No alcohol during outpatient feeding
- Participants will abstain from alcohol for 48 hours prior to Period B

- Caffeine containing products (with nothing added) will be permitted during the study with the following restrictions: caffeine containing products may not be consumed within 72 hours prior to admission.
- Participants will consume beverages of your choice that are calorie free such as water, black coffee (no cream/milk, sugar or artificial sweeteners), or other calorie-free beverages without artificial sweeteners such as unsweet iced tea, herbal tea, or black coffee. If you have other beverage preferences, please speak to study staff
- Use of tobacco and nicotine-containing products is not permitted in this study
- Participants will abstain from strenuous exercise (eg, heavy lifting, weight training, calisthenics, aerobics) for at least 48 hours prior to each admission. Walking at a normal pace will be permitted
- Participants will be instructed to maintain usual physical activity in between visits

Children: We will aim to recruit men and women between the ages of 18 and 45; therefore, we will not recruit children in this study.

Women: We will aim to recruit balanced numbers of men and women in this study. Our recruitment efforts in previous studies have recruited at least 50% women.

Minorities*:* According to the 2012 census, the average population of the greater Orlando Tri-county (Orange, Seminole, and Osceola) is slightly over 1.9 million people. Of these, 0.60% are America Indian/Alaska Native, 4.10% are Asian, 0.17% are Native Hawaiian/Pacific Islander, 15.53% are Black or African American, and 77.23% are White. Approximately 31.40% are Hispanic. For this study, we plan to recruit participants proportionate to the overall makeup of the community.

Resources Available
Site Initiation / Study Specific Training

We attest that all TRI faculty and staff will be trained and this training will be documented. We will adhere to ADVENTHEALTH ORLANDO ORA SOP 06 (Research Personnel Selection, Qualification, Responsibilities, and Training) and POLICY-TRI-QM-002 (Training Policy).

Study Procedures

Participant Recruitment and Screening
Recruitment methods utilized may include, but will not be limited to, the following: recruitment from within the TRI-MD's patient population: electronic medical records and database searches (including third party recruitment vendors); advertising in multiple media such as print ads, flyers, brochures, posters; radio ads; television spots; and internet advertising. All advertising materials will be submitted to the IRB for review prior to using or publishing them.

Recruitment efforts will follow ADVENTHEALTH ORLANDO recruitment SOPs for research. AdventHealth Orlando Employees will not be individually targeted nor excluded from study participation based on employment. ADVENTHEALTH ORLANDO employees who

engage the TRI-MD asking to participate in the study will be processed per standard consent procedures for participants. In addition, during the consent process, the study staff will review standard consent language stating that an employee's participation or lack of participation in the study will not affect their employment status or relationship with AdventHealth Orlando.

Consent Process and Documentation
We attest that all study staff delegated the authority to obtain informed consent will follow "Investigator Guidance: Informed Consent (HRP-802)", as well as "Investigator Guidance: Documentation of Informed Consent (HRP-803)".

Non-English Speaking Participants

N/A

Waiver of Written Documentation of Consent or Waiver of Consent
N/A

Documentation of Informed Consent Process
Documentation of the informed consent process is required to establish that the participant was accurately and adequately informed and that no study-related procedures were initiated prior to obtaining informed consent. A research team member will note in the source documentation the consent process, date consent was obtained and that consent was obtained prior to initiating any research procedures.

Randomization
This is a crossover design where all participant will consume both diets in random order. Randomization will be determined by the TRI statistician.

Study Visits
All study visits will be conducted at the Translational Research Institute for Metabolism and Diabetes.

Clinical Study Design

Screening Visit, Day -28 to Day -1 (outpatient, ~2 hours): Participants will arrive for this outpatient visit after an overnight fast. After obtaining informed consent, data collection for assessing eligibility will commence. The screening visit will include a urinalysis, screening blood work (including CBC with differential, comprehensive metabolic panel, HbA1C, TSH), pregnancy test for women of childbearing potential, drug screening, a complete medical history (including, but not limited to, alcohol use, concomitant medications, health conditions, gestational age/birth weight (if available), diet preferences/allergies and exercise habits), Antibiotic Use Questionnaire, Bowel Health Questionnaire, physical exam and anthropometrics (vital signs, height, weight, BMI, waist to hip ratio). Participants, who are eligible based on this evaluation will undergo an orientation session, introduction to the clinic, CRU and scientific

staff. Upon review of all laboratory based screening endpoints, eligible participants will then be scheduled for Day 1.

Visit 1/ Day 1 (outpatient, ~2 hours) +/- 2 Day window**:** Participants will arrive for this outpatient visit and will have the following performed: DEXA (Dual energy X-ray absorptiometry) and accelerometry to measure habitual activity as follows: armband accelerometer placed on the back of the upper left arm and wrist accelerometry. If scheduling challenges arise, DEXA can be done +/- 2 Days. Participants will wear the accelerometers to measure physical activity for 7 days with a goal to collect a minimum of 4 days of data. Physical activity will be quantified daily using high sampling frequencies with these small, non-invasive, portable accelerometers. This data will be used to determine the energy cost of exercise to predict total daily energy expenditure (TDEE) so that the calorie level can be set for the outpatient diet. Diet randomization will occur at this visit.

A stool sample will be collected before the start of the outpatient study diet. This sample needs to arrive at TRI on frozen cold packs within 24 hours of collection. Participants will be asked to provide the stool sample on Visit 1 (Day 1). If participant is not able to produce a sample, they will be provided with a stool specimen collection kit that includes all necessary supplies to collect the stool sample, a box with cold packs for transport and instructions for collection. Participants can collect the stool sample at home anytime between day 1 and day 9. Once produced, participants will call the study coordinator to make arrangements for the sample to be dropped off. If participant is unable to drop off the stool sample, a courier will be set up to pick it up from their home. If the stool sample is produced on Day 7, participant may bring it with them with the frozen cold packs to Visit 2 on Day 8. If the sample is produced on day 9, the participant may bring the sample in during Visit 3 on Day 10. This stool sample will only be collected during Period A. If not produced, the participant may continue in the study.

Visit 2/ Day 8 (outpatient, ~ 30 minutes) +/- 2 Day window**:** Participants will return to the clinic to return their accelerometers. Overall physical activity levels, daily changes, amount of time spent in sedentary/ moderate/vigorous intensity categories and activity-associated energy expenditures will be extracted. This data will be used to set the calorie level for the outpatient diet.

Visit 3-6 Day 10-20/ Visit 9-12 Day 39-49 (outpatient/inpatient): Outpatient controlled diet: Participants will return to the clinic and they will be provided with either a typical Western diet or an ME diet to consume. They will consume this diet for a total of 11 days on an outpatient basis except for Days 11-13/40-42 when they will be inpatient; see below. Participants will be asked to consume 100% of all food given. Participants will check their weight at home with a digital scale that will be provided to them. Participants will return to the clinic up to 3 times per week to consume breakfast on site and take home meals to consume for the number of days until their next clinic visit. During the clinic visits, study staff will assess compliance and tolerability of diet. Any food that is not consumed will be weighed back.

Note that Visit 8, Day 38, removed from protocol and subsequent visit titles will remain as listed.

On Day 10/39, polyethylene glycol (PEG) will be administered at a dose of 0.5 g (in a gelatin capsule) TID to the end of the calorimetry test period (Day 29/58). The goal of this diet period is to adapt the gut microbiota to the diet.

Day 11-13/ Day 40-42 (inpatient): On Day 11/40, participants will be admitted to the TRI Clinical Research Unit after dinner and stay overnight before entering the chamber on day 12/41. They will continue their diet throughout their stay. On Day 12/41, we will determine energy requirements for their subsequent inpatient stay with whole room calorimetry. In the chamber, the participant will perform the same activity schedule including rest, meals, treadmill walk, desk time and sleep that they will perform on Days 24-29/53-58. They will also wear armband and wrist accelerometers. They will be discharged on Day 13/42 and they will continue their outpatient diet.

Visit 7 Day 21-32/ Visit 13 Day 50-61 (inpatient): Participants are admitted into the metabolic ward for an 11-day period of in-depth quantitative phenotyping and to determine his/her individual energy requirements in a whole room calorimeter using a previously validated method (see ClinicalTrials.gov NCT01967563 and Preliminary data in grant). As per our standard methods, there will be a schedule for meals and for activity that is similar while in and out of the calorimeter. Participants will continue PEG dosing and will continue on the same diet assignment as the preceding outpatient diet phase.

Day 21-23/ Day 50-52 (inpatient): Period of participant adaptation to the ward and its routine. Upon admission and throughout the inpatient stay, vital signs and adverse events will be monitored. On day 21/50, weight will be checked. Participants will become aware and accustomed to the unit and its routine; included, but not limited to wake up/sleep times, diet times, clinical monitoring (weight/blood draws/medication administration, etc.) and food/VAS questionnaires. At Day 22 /51, we will collect information on usual diet (Diet History Questionnaire II). On Day 23/52, a DEXA and MRI/S will be conducted. If scheduling challenges arise, DEXA and MRI/S can be done +/- 2 Days. Hemoglobin will be checked on Day 52 to assess whether the hemoglobin allows for another round of blood collection on Day 59. If hemoglobin levels are <12 g/dl in men, <11 g/dl in women, blood collection timing will be reassessed to ensure safety.

Day 24-29/53-58 (inpatient, whole room calorimetry): Six-day period of data-collection in the whole-room calorimeter. Volumes of O_2 consumed and CO_2 and CH_4 produced (VO_2, VCO_2, and VCH_4 respectively in L/d) will be measured continuously. During the calorimeter stays, food presented will be based on the previously measured total daily energy expenditure to achieve energy balance. Day 24/53 energy intake (EI) will match energy expenditure (EE) measured during the Day 12/41 whole room calorimeter stay. Day 25/54 EI will match EE from Day 24/53, Day 26 EI will match Day 25/54 EE and this pattern of adjusting intake to match the previous day's expenditure will continue through Day 29/58.

Duplicate meals will be prepared, homogenized, and analyzed for energy and macronutrient content on each of the 6 calorimeter days.

On one of the six calorimetry days, participants will swallow a pH-sensing orally ingested radio-transmitter pill to measure the pH and therefore GI transit times. Participants will have the radiotransmitter pill administered on the same calorimetry day during Period B.

Participants will be asked to consume 100% of all food given. If unable to do so, unconsumed food will be weighed and matching units will be provided to achieve energy balance.

All fecal matter will be collected over 24-h for 6 days and either aliquoted for specific endpoints or composited to measure stool-related endpoints including: fecal energy, extract DNA and RNA for microbial community structure and function, and calculate nutrient absorption (as the % of ingested calories). Participants will be allowed to exit the calorimeter for a shower for 1 hour each day; however, all urine samples and fecal material will be collected - if produced - while outside the calorimeter.

Urine will be collected for each 24-h period, and nitrogen/creatinine will be measured for the calculation of substrate (fat, carbohydrates and protein) oxidation rates as well as nonprotein respiratory quotient (npRQ). Creatinine will be used to normalize the data and as a quality control check for completeness of collection.

Activity in the calorimeter will be regimented based on a previously developed and validated protocol [ClinicalTrials.gov NCT01967563]. This lessens the effect of day-to-day variation in Non Exercise Activity Thermogenesis/Spontaneous Physical Activity can have on total daily energy expenditure. This regimen provides greater accuracy on RQ and changes in RQ (a.k.a. metabolic flexibility). The calorimeter regimen also allows us to calculate resting EE, thermic effect of feeding (TEF), activity EE (treadmill, fixed pace and incline), and Sleep EE (SEE).

On Day 58, if the Hemoglobin at Day 52 does not meet criteria, a repeat will be performed stat before the blood is drawn on Day 59.

Day 30/59 (inpatient, enteroendocrine testing): Participants will have an IV inserted to collect fasting blood to analyze for microbial products such as SCFA and enteroendocrine hormones. Blood enteroendocrine hormones will be measured (total of 18 blood samples over 24h) pre and post meals to gain insight into the effects of inter-individual microbiome makeup and the ME diet on upper bowel incretin and satiety hormones (acyl and des acyl ghrelin, active GLP-1, and GIP), as well as distal satiety signals (PYY3-36). Sample collection will be as follows:

An antecubital IV will be inserted. Paired baseline (fasting/post-absorptive samples at nominal time point -30 and -15 before breakfast) samples will be collected for routine metabolic measures (lipids/free fatty acids, glucose, insulin, C-peptide) and hormones:

(leptin, des acyl and acyl-ghrelin, total and active GLP-1, CCK, GIP, and PYY 3-36). Meals will be provided at the same times as in the calorimeter and of the same composition as the assigned diet (ME vs. Western). Meals must be consumed within 20 minutes with a 10 minute window to account for rare instances of where 100% of meal cannot be consumed. Blood will be collected at -30, -15, + 30, +60, + 120, and +180 minutes pre/post each meal (x 3) for measurement of the same tests as the paired baselines for a total of 18 samples. AUCmeal and AUC24h will be calculated using a trapezoid method.

At the second baseline time point (nominal tine point -15 before breakfast), additional tubes will be collected for biobanking as follows: serum, plasma, and whole blood (for DNA and RNA). We will also collect an additional tube for DNA and RNA 12 hours after the second paired baseline sample.

The study site will provide a dose of 1.5 g acetaminophen at nominal time point zero (45 mL of liquid form for oral administration with concentration of 500 mg/15 mL), which will be administered orally as a challenge agent to assess gastric emptying during inpatient visit. Acetaminophen will be drawn into a syringe by the site pharmacist and dispensed to the inpatient CRU for administration to subject. The blood sampling for determining acetaminophen concentrations will be performed at each of the pre-defined nominal time points for the breakfast meal that are being used for enteroendocrine testing: -30, -15, +30, +60, +120, and +180 minutes.

All urine produced over 24-h will be collected on day 30/59 to be aliquoted for specific endpoints related to energy balance, metabolism and enteroendocrine hormones.

Day 31/60 (inpatient, food intake testing): The IV will be removed, and food-intake testing performed. This follows the standard paradigm for TRI: a fixed calorie-content breakfast (500 calories) using foods from the assigned diet. Then, a selection of foods, again from the assigned diet, in a quantity greater than needed for energy balance (1.5 X a typical meal), will be presented for lunch and dinner. These buffet meals will be served and then consumed *ad libitum* over 30 minutes in our food-intake lab (controlled temperature, limited access, and HVAC isolated from kitchen smells). Participants will be instructed to eat as much or as little as they like. Food items will be weighed pre and post meal and total energy intake (kcal) and macronutrient intake (g fat, CHO, and protein). Visual Analog Scales (VAS) will be administered at -30, -15, + 30, +60, + 120, and +180 minutes post each meal for hunger, a standard TRI procedure developed in collaboration with Corby Martin at the Pennington Center.

On Day 31/60, a DEXA will be conducted. If scheduling challenges arise, DEXA can be done +/- 2 Days.

The next morning (**Day 32/61**), participants will be weighed (fasting) and then provided a courtesy breakfast of their choice (pre-ordered while in the unit). Participants will then be discharged to either cross over into the other diet or their participation will be complete if they already went through both diet periods.

The crossover period will be up to 14 days.

<u>Please see table below for a Schedule of Activities.</u>

	SV	V1	V2	V3	V4					V5			V6		V7											
				V9	V10					V11			V12		V13											
	SV Day -28 to Day -1	Day 1	Day 8	Day 10	Day 11	Day 12	Day 13	Day 14	Day 15	Day 16	Day 17	Day 18	Day 19	Day 20	Day 21	Day 22	Day 23	Day 24	Day 25	Day 26	Day 27	Day 28	Day 29	Day 30	Day 31	Day 32
				Day 39	Day 40	Day 41	Day 42	Day 43	Day 44	Day 45	Day 46	Day 47	Day 48	Day 49	Day 50	Day 51	Day 52	Day 53	Day 54	Day 55	Day 56	Day 57	Day 58	Day 59	Day 60	Day 61
Informed Consent	X																									
Review of Eligibility	X																									
Food preference and allergy assessment	X																									
Demographics	X																									
Adverse Events/Con Meds	X	X	X	X	X	X	X			X			X		X	X	X	X	X	X	X	X	X	X	X	X
Vitals	X	X			X	X	X								X	X	X	X	X	X	X	X	X	X	X	X
Bowel Health Questionnaire	X																									
Medical History and Physical Exam	X																									
Anthropometrics- Height, weight, BMI, waist to hip ratio	X																									
Antibiotic Use Questionnaire	X																									
Urinalysis/Toxicology Screen	X																									
Pregnancy Test (serum for SV; remaining tests are POC urine)	X	X															X								X	
Screening Labs	X																									
Diet History Questionnaire (Period A Only)																X										
Activity History	X																									
Arm/wristband Accelerometer Placement		X				X																				
Arm/wrist band Accelerometer Return			X				X																			
DXA		X															X								X	
Randomization to Diet		X																								
Dispense Home Stool Collection Kit (Before Period A ONLY)		X																								
Whole Body MRS - visceral vs. subcutaneous, hepatic fat and organ size																	X									
Run-in Calorimetry						X																				
Hemoglobin Check (Day 52 and if needed Day 58 ; see protocol)																	x						x			
Outpatient Controlled Diet				X	X		X	X	X	X	X	X	X	X												
Inpatient Controlled Diet					X	X	X								X	X	X	X	X	X	X	X	X	X	X	
Breakfast on site with meals sent home for remaining days				X			X			X			X													
PEG Dosing				X	X	X	X	X	X	X	X	X	X	X	X	X	X	X	X	X	X	X	X			
Home weight recording				X				X	X		X	X		X												
In clinic weight check					X	X	X			X			X		X	X	X	X	X	X	X	X	X	X	X	X
24 hour Whole Room Calorimetry						X												X	X	X	X	X	X			
24 hour Fecal Collection																		X	X	X	X	X	X			
24 hour urine						X												X	X	X	X	X	X	X		
Radiotransmitter Pill Administration (administration day any day during calorimetry)																		X								
Enteroendocrine Testing																								X		
Acetaminophen Administration																								X		
Standardized Breakfast																								X		
VAS Pre and post meals																									X	
Fixed Breakfast																									X	
Buffet Meal (lunch)																									X	
Buffet Meal (dinner)																									X	
Courtesy breakfast																										X
Admit					X	X									X											
Discharge							X																			X
Overnight Stay					X	X									X	X	X	X	X	X	X	X	X	X	X	

Analytical / Clinical Procedures

Specific Clinical Methods for Aim 1:
Medical History and Physical Exam
A comprehensive health history will be obtained. A standard physical examination will be performed by a study physician, physician assistant, or nurse practitioner.

Anthropometric Screening
Body weight (calibrated scale), height, waist and hip circumference will be obtained while in a gown/scrubs, without shoes. BMI will be calculated.

Home Weight Checks:
Participants will receive a Tanita digital scale for daily weight checks. Weights will be documented on a weight diary provided by TRI-MD.

Screening Labs
Labs will be collected as described previously and total blood volume collected will be approximately 9 ml.

Vital Sign Measurements
Rested measurement of vital signs will include heart rate (HR), blood pressure (BP), respirations and temperature.

Measurement of energy expenditure and methane production rates in a whole room calorimeter
Whole room indirect calorimetry allows for the simultaneous measure of total energy expenditure (kcals/min) and substrate oxidation (RQ) in a free living environment with multiple activities. Room calorimeters provide greater precision in the measure of oxygen consumption and carbon dioxide production as they provide a controlled testing environment and use more robust gas analyzers. Fat oxidation will be highest during periods of low activity [and fasting] as the metabolic system switches to relying on fat stores as the main energy source. Fat oxidation decreases as metabolic rate (kcal/min) increases through muscle activity.

Installed and validated in 2012, the two "large room" TRI-MD calorimeters measure 10 feet x 12 feet x 9 feet with a total volume of 31,000 L. They feature state-of-the-art technologies in a push – pull configuration. The calorimeter will be maintained at thermoneutrality 75°F/22.5°C and a relative humidity of 50-65%. The chamber is ventilated with medical air at a rate controlled by the chamber software to maintain CO_2 levels ~0.4% with a range of 20-90L/min. The room has two windows, and is furnished with a bed, desk and chair, television, sink and toilet with privacy curtain, a treadmill, a chilled air-locking pass-through for the storage of urine and fecal samples, and an air-locking food pass-through for meal delivery. Video cameras and microwave motion detectors continuously monitor the participant's movement. Oxygen and carbon dioxide levels in the chambers are measured using a SIEMENS Ultramat/Oxymat 6 (SIEMENS, Bartlesville, OK) which sample O_2 and CO_2 concentrations in parallel 60 times per second. Every five seconds a computer program averages these values (15, 30, 60 second averaging capabilities),

calculates the volumes of O_2 consumption and CO_2 production, and plots the average values at 1-minute intervals. On every test day the chambers are calibrated using pure gas mixtures, and for determination of the accuracy and precision of the calorimeters, short circuit gas infusions are performed weekly, 23-hour gas infusion tests are performed based on chamber availability, maximum time between tests is 3 months. The accuracy of our chambers is 99.77 % and 99.56 %, for O_2 and CO_2, respectively. Energy expenditure and substrate oxidations (including RQ) are calculated from O_2 consumption, CO_2 production and 24-hour urinary nitrogen excretion by using the equations established by Weir. Input air consists of 100% dehumidified medical air that is buffered through two compressed air tanks.

Brief overview of daily calorimetry routine: The participant will void prior to entry into the chamber. At this point, a timed urine collection will begin for the duration of the stay in the chamber. Participants will follow a set routine in the calorimeter that matches the activity routine in the CRU acclimatization period. Rest, meals, treadmill walking, desk time, and other activities are scripted to the minute and observed by the staff of the CRU and the Energy Expenditure Core via camera. The energy cost of activity is determined during the screening process. Conditions during the stay will be standardized based on TRI's standard procedures (NCT 01967563). Inflow and chamber concentrations of O_2 and CO_2 are measured continuously, as are activity and environmental conditions and the rate of air flowing in to and out of the calorimeter via high accuracy mass flow controllers (MFCs). Urinary Nitrogen and Creatinine are measured over 24 hours for all days in the calorimeter. These values are fed into a computer system (PiLR™) that stores the data, calculates VO_2, VCO_2, and VCH_4, and converts these into EE (kcal/minute) and RQ output every minute. Combined, fat, carbohydrate, and protein oxidations will be calculated as per Elia and Livesey and our recent publication in the American Journal of Clinical Nutrition.

A novel feature of this study is that we will measure methane production over 24 hours so that it can be compared to the gut microbial ecology (by qPCR and RNASeq) and accounted for energy balances. To achieve this, the TRI-MD calorimeters will be upgraded to sample the outflow gas via an additional MFC into a specialized gas chromatograph (GC) for the measurement of methane. Methane can be assumed to be produced at a rate of approximately 1 to 3L / day, but has never been directly measured in humans. Given the calorimeter volume of 31,000 L, we calculate that methane concentrations over the 23 h will start at approximately 1.8 ppm (nominal air methane concentrations) and finish at ~100 ppm. MEI, Inc., the engineering company who designed and installed the calorimeters, will write code to incorporate the calculation of VCH_4 into our software. Furthermore, using the pure-gas blender system MEI developed and installed at TRI-MD, we will validate the system via injections of methane into the calorimeter at fixed rates - all production rates then being traceable back to NIST standards.

These experiments will be conducted with the expertise of a collaborative engineering team (MEI, TRI-MD, and ASU). Furthermore, the first two participants will be run to test and validate these calorimeter systems. VCH_4 will be input into the energy balance model and will also be compared to the quantitative PCR (qPCR) data measuring the abundance of methanogens in the fecal matter.

Prediction of energy needs in a whole room calorimeter.

We have recently published a paper on this technique. Also, please see the procedure below for calculations on TDEE estimates for outpatient meals.

Maintenance of energy balance in a whole-room calorimeter.

The arm/wristband measured free living energy expenditure will be combined with an algorithm to calculate an initial prediction for the single Day 12/41 chamber stay. The measured energy expenditure from the Day 12/41 chamber stay will determine the base calorie level for the 6 day run of calorimeter stays (Days 24-29/53-58). A portion of the menu will consist of 50 kcal units that will allow for minor adjustments from day to day. Day 24/53 intake will be exactly what was measured and Day 12/41 but each subsequent day's EI will be based on the previous day's measured EE for the remaining 5 chamber stays. If EE changes compared to the prediction, 50 kcal units will be added or removed to match the change. This method combined with the consecutive days of controlled activity will allow us to achieve energy balance within ± 50 kcal for the totality of 6 calorimeter days.

24 hour urine collection

During each 24 hour respiratory chamber stay, all urine will be collected for measurement of total nitrogen and creatinine. During day 30/59, urine will be collected for specific endpoints related to energy balance, metabolism and enteroendocrine hormones.

24 hour fecal collection

Fecal samples will be collected during the 24 hour chamber stays during the diet periods by placing disposable commode specimen containers under their toilet seats before bowel movements. Participants will be given daily doses of polyethylene glycol or PEG (e.g. Miralax) 500 mg, three times a day with meals, as continuous marker to accurately measure the absorption of nutrients from the intestines. Samples will be stored at ≤ −20°C until further analysis. These analyses include, but are not limited to: COD, short chain fatty acids (SCFAs), and sugars.

Dual energy x-ray absorptiometry (DEXA)

DEXA scans will be performed using the General Electric Lunar iDXA (General Electric; Milwaukee, WI, USA) to determine body composition. Participants will change into a hospital gown/scrubs, remove all metal-containing objects and lie on a table while the scanner, which emits low energy X-rays while a detector passes along the body. The scan will take approximately 15 minutes.

Gastric Emptying

Gastric emptying will be assessed using plasma acetaminophen concentrations over 3 hours. After fasting blood sampling on Day 30/59, participants will receive liquid oral acetaminophen 1.5 g with a standardized breakfast , which must be consumed within 20 minutes with a 10 minute window to account for rare instances of where 100% of meal cannot be consumed. The blood sampling for determining acetaminophen concentrations will be performed at each of the pre-defined nominal time points for the breakfast meal that are being used for enteroendocrine testing: -30, -15, 30, 60, 120, and 180 minutes.

Diet History Questionnaire
A food frequency questionnaire (National Cancer Institute Diet History Questionnaire II) will be administered to all participants. The National Cancer Institute Diet History Questionnaire II is a self-administered, web-based (http://epi.grants.cancer.gov/dhq2/) questionnaire consists of 134 food items and 8 dietary supplement questions that assess a comprehensive list of nutrients. The original paper version of this questionnaire was extensively validated and the current version is expected to provide comparable results. Data will be analyzed with DietCalc Software (National Cancer Institute). Participants will be provided with an ID and password to log into the secure, web based questionnaire. The questionnaire takes about 1 hour to complete.

Physical activity monitoring
Physical activity will be quantified with activity monitors using high sampling frequencies during a 7 day period after screening, using a small, portable armband and wristband accelerometers. We will also quantify energy expenditure during the first calorimeter stay in both study periods. Overall physical activity levels, daily changes, amount of time spent in sedentary, moderate, vigorous intensity categories and activity-associated energy expenditures will be extracted. Two triaxial activity monitors will be used. One is an armband that is wrapped around the upper left arm (SenseWear Pro3 Armband, BodyMedia Inc.). The other is worn around the wrist (Actigraph GT3X, Actigraph LLC). The monitors integrates motion sensor data with a variety of heat-related sensors to estimate the energy cost of free-living activity. Two accelerometers are being used because the SenseWear armband is not being manufactured anymore. Although we have sufficient stock, the second accelerometer will allow us to cross validate the wrist accelerometer while assuring that if we have a problem with the armband, we are still able to get the data needed for the study. The participant will wear the monitor for 7 days during period A (Days 1-7) and on calorimeter days 12/41, except while showering or bathing. The participant will have this monitor removed and the data uploaded at study Day 8 and 13/42.

Magnetic Resonance Imaging and Spectroscopy
The goal of this test is to assess lipid content, volumetric fat and organ volume quantitation using an Acheiva 3T (Philips, Amsterdam, the Netherlands).

Intrahepatic Lipid (IHL) content will be measured using both imaging and spectroscopy. Scans will be performed under standardized conditions with participants in a supine position. Standard clinical MR Imaging, including 3-plane localization and T1 weighted images, will be completed to obtain anatomical images for voxel / slice localization. A 1H quadrature body coil (QBC) and torso XL coil will be used to measure intrahepatic fat stores. A single voxel point resolved spectroscopy (PRESS) sequence will be acquired from a 2 x 2 x 2 cm3 volume in the upper right lobe of the liver in an area that is free from heavy vascularization as determined from the scout images.

Proton Density fat fraction imaging sequences will be performed covering at least the entirety of the liver.

Volumetric measurement of fat, muscle, organs and bone will be completed across the whole body. Scans will be performed under standardized conditions with participants in a supine position using

the quadrature body coil and torso XL. Low resolution scans will be completed to determine appropriate positioning for high resolution images.

Total MR scan time will be approximately 90 minutes.

Resultant images will be analyzed using Analyze 11.0 (Biomedical Imaging Resource, Mayo Clinic, Rochester MN) to segment depots of fat (e.g. subcutaneous vs. visceral) as well as bone and muscle volume. Spectroscopy will be analyzed using jMRUI (the MRUI Consortium).

Diet - Design Principles for Microbial Enhancement.

We designed the ME diet as a tool to modify the gut microbiota (only for research purposes; not as a suggested diet for weight loss) by surveying the available literature and identifying four key drivers that should increase the amount of food that enters the colon:

1. foods that are rich in dietary fibers, including whole grains, whole wheat, fruits, and vegetables;
2. foods that resist particle-size reduction, including nuts and oats;
3. foods that are high in resistant starch, e.g., bananas and jicama; and
4. less-processed foods, such as whole fruits instead of fruit juice, steak instead of ground beef or processed meats, and yogurt with almonds and granola instead of yogurt with added simple sugars. The table below provides additional details:

Table 1: Microbiome Enhancer Diet Drivers

Drivers	Approach	Desired Effects	Potential Drawbacks
**1. Increase fiber**	Nutrient substitution (e.g., use whole grains instead of white grains)	Increase carbohydrate delivery to the colon	gastrointestinal symptoms
**2. Increase amount of foods that maintains a large particle size**	Nutrient substitution (e.g., use nuts or quinoa as protein source instead of meat)	Increase loadings of dietary fibers and proteins associated with dietary fibers.	Association of proteins and dietary fibers can confound mechanistic description (co-variance)
		Proteins may mitigate the risk from inconsistent starch absorption	Change the forms of fat that is available to the colon (co-variance).
		Decrease upper GI absorption	
**3. Increased resistant starch**	Nutrient substitution	Increase carbohydrate delivery to the colon	Inconsistent starch absorption
	Food preparation (e.g., cold potato salad more resistant than boiled or baked potato; relatively unripe banana)		
**4. Less processed food**	Food preparation (e.g., Steak versus ground meat)	Departure from carbohydrates	Proteins will have smaller impact than carbohydrates on the stool energy differences.
		Testable with bioreactors	
		Stimulate protein utilizing microbiota	
		Lower the importance of upper GI absorption	

Example menus are shown below for the purposes of illustrating the types of foods in each diet and particularly, the implementation of the ME Diet Drivers. Each diet will be fed on a minimum three-day rotation. Participant ability to consume study foods will be assessed prior to enrollment.

Table 2: Example Menus

Western Menu 1	Microbiome Enhancer Menu 1
Breakfast<br>• Grape Juice, 4 fl oz (126.4 g)<br>• Cereal, Corn Flakes, 1 cup (28 g)<br>• Sugar, 1 packet (2.8 g)<br>• Bread, white, toasted, 1 slice (25 g)<br>• Margarine, soybean, 2 tsp (9.4 g)<br>• Milk, 2% fat, 1 cup (244 g)	Breakfast<br>• Orange, fresh, large (151 g edible portion)<br>• Cereal, Kashi Go Lean Honey Almond Crunch (53 g)<br>• Whole wheat bread, 100%, 1 slice (28 g)<br>• Butter, 1 pat (5 g)<br>• Milk, 2% fat, 1 cup (244 g)<br>• Pear, raw, 1 medium (178 g edible portion)
Lunch<br>• Turkey sandwich<br>○ Deli turkey, 3 oz (85 g)<br>○ Mayonnaise, 1 pkt (12 g)<br>○ Lettuce leaves (15 g)<br>○ Bread, white, 2 slices (50 g)<br>• Dill pickle spear (30 g)<br>• Potato chips, baked, 1 oz (28.35 g)<br>• Strawberry nonfat yogurt with artificial sweetener, 4 oz (113.5 g)	Lunch<br>• California-style sandwich<br>○ Zucchini squash, broiled, ¼ cup (45 g)<br>○ Avocado, raw (50 g)<br>○ Cheese, Swiss (21 g)<br>○ Spinach, raw (40 g)<br>○ Tomato, raw, slices (40 g)<br>○ Olive oil, 2 tsp (9 g)<br>○ Multigrain bun (43 g)<br>• Banana, raw, small (101 g edible portion)<br>• Carrots, baby (50 g) and celery sticks (85 g) with light ranch dressing, 1 tbsp (15 g)
Dinner<br>• Italian dinner menu<br>○ Ground beef, 10% fat, cooked, 5 oz (142 g)<br>○ Spaghetti sauce, Prego (100 g)<br>○ Spaghetti, plain, cooked, 1 cup (140 g)	Dinner<br>• Italian dinner menu: top sirloin steak with a side of spaghetti with sauce<br>○ Beef, top sirloin, separable lean, trimmed to 0" fat, 3 oz (85 g)<br>○ Spaghetti sauce, Prego (100 g) with spaghetti, whole wheat, cooked, 1 cup (140 g)<br>○ Cheese, parmesan, 1 tbsp (5 g)

○ Cheese, parmesan, 1 tbsp (5 g)<br>• Cauliflower, cooked, pieces, ½ cup (62 g)<br>• Dinner roll, brown & serve, 1 roll (28 g)<br>• Margarine, soybean, 1 tsp (4.7 g)<br><br>• Snack or Dessert for Dinner meal<br>• Cake, pound, without icing, 2 oz (56 g)<br>• Lemonade-flavor drink powder (18 g) – to be mixed with water	• Broccoli, cooked, 3 spears (111 g)<br>• Roll, oat bran dinner roll, 1 (33 g)<br>• Butter, 1 pat (5 g)<br>• Jicama salad with cucumber and lime<br>○ Jicama, raw, ½ cup slices (60 g), cucumber with peel, ½ cup slices (52 g), lime juice, ½ oz (15.4 g)<br><br>• Snack or Dessert for Dinner meal<br>• Strawberries, raw, 1 cup halves (152 g)<br>• Topping for strawberries: yogurt, plain, low fat, high protein (2 oz), with almonds, ½ oz (14.2 g) and granola, Quaker 100% Natural Granola Oats and Honey (20 g)

Western Menu 1<br>Nutrient Values		Microbiome Enhancer Menu 1<br>Nutrient Values	
• Kilocalories	2039	• Kilocalories	2043
• Protein, g	90.3	• Protein, g	92.4
• Protein, % energy	17.7	• Protein, % energy	18.1
• Fat, g	70.7	• Fat, g	71.8
• Fat, % energy	31.2	• Fat, % energy	31.6
• Carbohydrate, g	259.7	• Carbohydrate, g	280.6
• Carbohydrate, % energy	50.9	• Carbohydrate, % energy	50.3
• Total Dietary Fiber, g	12.8	• Total Dietary Fiber, g	57.2

By Meal Information:	Western Menu 1	Microbiome Enhancer Menu 1
Breakfast, kcal	445	600
Fat, g	13.5	13.4
Protein, g	12.6	22.7
Carbohydrate, g	70.3	103.9
Total Dietary Fiber, g	1.4	19.1
Lunch, kcal	517	542
Fat, g	19.0	29.4
Protein, g	21.0	14.9
Carbohydrate, g	65.5	61.4
Total Dietary Fiber, g	3.4	12.5
Dinner, kcal	791	644
Fat, g	29.2	16.7
Protein, g	53.0	45.6
Carbohydrate, g	76.6	83.5
Total Dietary Fiber, g	7.6	19.5
Snack/Dessert, kcal	286	257
Fat, g	9.0	12.3
Protein, g	3.6	9.1
Carbohydrate, g	47.4	31.7
Total Dietary Fiber, g	0.3	6.2

Diet - validation procedures:

Diets designed using these four principles will be prepared, composited, and sent for food analysis. Revisions to the diets will be made to achieve calorie and macronutrient targets that are balanced across two diets.

Out-patient diet procedures.

Participants will be fed breakfast at the TRI-MD in the dining room up to 3 times per week, while lunch and dinner will be packed to go. Calories will be given based on average free-living EE (armband accelerometer; previously validated against doubly labeled water) at a level of 15% greater than estimated TDEE.

In-patient diet procedures.

Participants will be fed meals in the TRI-MD, in the dining room or calorimeter. Given that quantifying energy balance is a primary goal the study all meals will be monitored by CRU staff at regular intervals with provision of reminders, encouragement, and removal of

barriers/distractions to ensure compliance.Participants will be asked to consume 100% of meals. In the event this does not happen, food not consumed will be returned to the kitchen, weighed and recorded. In the calorimeter, 50 calorie units will be provided to achieve energy balance.

~~~~~~~~~~~~~~~~~~~~~~~~~~~~~~

Specific Clinical Methods for Aim 2

<u>Relative Gastric Emptying:</u>
Orally administered acetaminophen has been used extensively to assess gastric emptying in humans. Orally administrated acetaminophen is rapidly absorbed by the small intestine, but not by the stomach. The acetaminophen method correlates well with scintigraphic measurements without radiation exposure.

<u>Enteroendocrine testing:</u>
Blood enteroendocrine hormones will be measured (18 samples over 24h) pre- and post-meals to gain insight into the effects of inter-individual microbiome and the ME diet on upper bowel incretin and satiety hormones (acyl and des acyl ghrelin, active GLP-1, CCK and GIP), as well as distal satiety signals (PYY3-36).

On Day 30/59, an antecubital IV will be inserted. Paired baseline (fasting/post-absorptive) samples will be collected for routine metabolic measures (lipids, glucose, insulin) and hormones. Meals will be provided at the same times as in the calorimeter and of the same composition as the assigned diet (ME vs. Western). Blood will be collected at -30, -15, + 30, +60, + 120, and +180 minutes post each meal (x 3) for a total of 18 samples. AUCmeal and AUC24h will be calculated using a trapezoid method.

Blood volume collected per phase, including all additional hemoglobin checks, will not exceed 371 ml per phase.

<u>Radiotransmitter:</u> Participants will be asked to swallow a small, silicone radiotransmitter that sends data wirelessly to a sensor worn by the participant to record internal temperature, pressure and pH to assess gastric emptying. The capsule normally remains in the body for 24-72 hours. Fecal samples will be collected until radiotransmitter pill has been retrieved.

<u>Food intake / VAS:</u>
On Day 31/60, participants will be fed a 500 kcal breakfast. For lunch and dinner, they will be presented with a buffet of foods from the assigned diet (1.5 X the energy content of their energy balance diet) and instructed to eat as much or as little as they like. Unconsumed food will be weighed to calculate total kcal consumed.

Participants will be asked to complete a survey to identify their perception of hunger (i.e., visual analog scale [VAS]) line with qualifying statements such as “Not at all”/“The least I can possibly” and “Extremely”/“The most I can possibly”, anchoring the line on the extreme left and
~~~~~~~~~~~~~~~~~~~~~~~~~~~~~~

right side, respectively. In response to each question, participants will be asked to draw a vertical mark on the horizontal line to represent the magnitude of their response to the question. A value for each response is quantified by measuring the distance of their mark (in mm) relative to the left end of the line. Therefore, the values (or "scores") for each question range from 0 to 100.

VAS scales will be administered at -30, -15, + 30, +60, + 120, and +180 minutes pre/post each meal for hunger, satiety, fullness as routinely performed at TRI-MD.

All foods will be weighed on electronic balance scales in gram weights to the nearest gram; foods consumed are determined by subtraction of food weights after the participant has eaten, from initial weights. Food energy and macronutrient consumption will be calculated using commercially available software. Outcome variables will be total caloric and gram weight intake and macronutrient distribution. Diet energy density score will be calculated as kcal of food consumed divided by grams of food consumed (kcal/grams).

During the meal testing, participants will be monitored, from a control room either via a one-way mirror or video camera, for safety and compliance with instructions. This will ensure that food items are being consumed and not disposed of in some other way. No video recording of the meal testing will be made.

Study Duration:
The overall study duration is approximately 90 days: a 28-day screening period (inclusive of wait time), a 3 day period of testing to set inpatient calorie levels, and two diet intervention periods: the first is 32 days and the second is 23 days with a period of up to 14 days in between (this period may be extended- see risk section).

Materials of Human Origin: Collection, Preparation, Handling and Shipping
All biological materials will be obtained per the procedures described in this protocol. For all of the study procedures, Study Specific Procedures (SSPs) will be prepared or existing SOPs will be used. Samples will be collected and analyzed according to the SSPs and techniques established at the TRI-MD laboratory and Arizona State University.

Biological materials will be stored at the TRI-MD. Access to samples is limited to designated study team members as indicated on the delegation of authority log. Biological materials will be maintained in a locked and secured area within the TRI-MD facility. Physical access is limited by badge swipe assignments.

Biospecimens collected for study-related endpoints, will be analyzed/tested at both AdventHealth Orlando and outside laboratories/institutions. These institutions may include Arizona State University, Pennington Biomedical Research Institute, Johns Hopkins, and ARL Laboratories. Additionally, remaining biospecimens will be archived for any additional hypothesis-related experimentation or testing **<u>for this study</u>**, which cannot be predicted at the time the protocol is developed.

Furthermore, if there are any left-over biospecimens after completing the above, any left-over biospecimens may be archived for the following: other research (**<u>not for this study</u>**), but **<u>consistent with the original aims</u>**. This other research can take place at AdventHealth Orlando or other institutions.

Lastly, a predetermined amount of biospecimen samples will be collected specifically for archiving for future use, such as other research (**<u>not for this study</u>**), but **<u>consistent with the original aims</u>**. This other research can take place at AdventHealth Orlando or other institutions.

The biospecimens collected for this study will be separated into biospecimen samples that will be used for the study and biospecimen samples that were collected to be archived for future use. After study aims have been achieved and study related endpoints have been measured and analyzed, any remaining biospecimens will be stored at the TRI-MD Laboratory Room 2404 and will also be considered as "archived biospecimens."

Access to samples is limited to designated study team members as indicated on the delegation of authority log. Biological materials will be maintained in a locked and secured area within the TRI-MD facility. Physical access is limited by badge swipe assignments.

Archived biospecimens will be used for any additional hypothesis-related experimentation or testing **<u>for the purposes of this study</u>**, **<u>consistent with the original aims</u>**, which cannot be predicted at the time the protocol is developed due to the evolving nature of scientific exploration.

Archived biospecimen samples may be stored indefinitely for future research. Archived biospecimens could be used for **<u>separate research</u>** by **<u>both</u>** AdventHealth Orlando scientists and scientists outside of AdventHealth Orlando. This would be allowed if the research **<u>is consistent with the original aims</u>** of this study and if they have scientific merit as determined by the Principal Investigator, with an additional review by the respective Program Director. For research outside of AdventHealth Orlando, a Material Transfer Agreement will be obtained, which will govern the transfer and chain of custody of the biospecimens outside of ADVENTHEALTH ORLANDO.

Study Outcomes Measures (Endpoints)

<u>Clinical Study endpoints</u>

Primary Endpoint

The <u>primary endpoint</u> for the protocol is the within-participant difference in fecal energy (via chemical oxygen demand, COD) normalized to the total daily energy intake and to the non-metabolizable marker PEG [COD (kcal) / PEG (g)].

Principal Secondary Endpoints

The <u>principal secondary endpoints</u> will test hypotheses about how changes in the gut microbiota might change enteroendocrine hormone secretion, hunger/satiety, and food intake.

To accomplish these aims and objectives we will complete the following tasks:

1. Quantify the microbial contribution to energy balance by:
   a. Defining critical microbiology parameters using bench-scale bioreactors (at ASU)

b. Under strict dietary and environmental controls (at TRI) we will measure or collect samples to assess:
   i. Nutrient Absorption [Primary Endpoint]
   ii. Energy balance with whole room calorimetry and a controlled diet, including an assessment of methane production, the loss of calories in the urine and energy expenditure VO_2 and VCO_2 [Secondary Endpoints]
   iii. enteroendocrine hormone secretion, hunger/satiety, and ad libitum food intake [Secondary Endpoints]
   iv. Bowel transit time
   v. 16S rRNA gene sequences
   vi. Fecal SCFA concentrations and composition

2. Utilize the clinical and laboratory data under objective 1.b. as inputs to develop and refine an *in silico* model of microbial ecology / metabolism (ASU).
3. Once the model is well developed, we will compare model outputs (predictions) to measured nutrient/energy absorption using state of the art metabolic techniques.
4. Create a cause-and-effect link between environmental parameters (i.e., diets of different composition) with quantitative measurements of human metabolism (at TRI) and detailed laboratory measurements of the gut microbial ecology (at ASU) including fecal constituents (chemical and microbiological).
5. Create a combined single model from microbiological biochemical and host models. The ultimate goal is to use the unified model to predict the effects of changes in one or more parameters on host energy balance and therefore risk of developing obesity or as a means of treating obesity.

Data Management and Quality Plan

Data De-identification

Participants will be enrolled using a Clinical Trial Management System (CTMS), which assigns each participant a unique participant identification number (PID). This PID is a code consisting of a combination of numerals and letters, which will serve as the identifier for this participant for this particular research study. Access to the link between PIDs and personal health information (PHI) and access to clinical data are only granted to the clinical research team as assigned on the Delegation of Authority log. All of the data will be entered in an identified fashion in our source documentation, which will then be stored in a de-identified (i.e. by the PID) in our case report forms used for analysis and in a secondary storage data warehouse . Both storage locations are secured and only assessable to the clinical research team. The link will not be used to re-identify participants except in the event of a serious adverse event (SAE) requiring "unblinding" to treat the participant. We will store the link in the CTMS and in a Clinical Research Database, where only the research team has access.

Data Confidentiality, Storage, and Retention

The identity and personal health information will be kept confidential to the extent permitted by the applicable laws and/or regulations and will not be made publicly available. If results of this study are published or presented, the identities will not be revealed. Confidentiality will be maintained during and after the study. This information is also included in our informed consent, which is discussed with participant prior to enrollment.

Study documentation and paperwork will be stored in our locked filing room. The data records will also be stored in as electronic records. This data will be safeguarded so that only those on the research team have access to any of the clinical data (both source documentation and data warehouse storage). The electronic data is maintained by Management Information Systems (MIS) security controls.

The data will be stored based on sponsor contract (i.e. usually 10 years). TRI-MD retention policy is maintained in our Records Management Policy. Electronic de-identified data will be kept indefinitely in our data warehouse.

Data Quality
Data quality control will occur according to our SOPs on Data Entry, Quality Control Procedures and Query Management. All data will be entered into electronic source documentation and checked against the paper source for accuracy by a second party (Data Entry SOP) and errors resolved through the Query Management SOP. Ten percent of the data points will be routinely checked at the beginning, middle, and close of a study for quality control (Quality Control SOP). Finally, all critical endpoints (as determined by the PI or Sub-I) will be assessed using quality control analyses. The data will be loaded into a Clinical Research Database. Data in the warehouse will also be routinely monitored over time.

Data Sharing (outside of AdventHealth Orlando)
Some of the endpoint testing will be conducted at outside laboratories/institutions. To perform these analyses/testing/etc. and to interpret results, certain data elements will need to be shared along with the biospecimen samples. Data Use Agreements (DUAs) will be obtained, which will identify the specific data elements to be shared and will govern the sharing of data related to this study. Data will be de-identified, but a link/code is managed within an electronic research management system and maintained by a study coordinator.

Should archived biospecimens be needed for research outside of AdventHealth Orlando and certain data elements that are connected to the archived biospecimen samples are needed to conduct the research, then Data Use Agreement(s) will be obtained. The Data Use Agreement(s) will identify the purpose for data sharing, the specific data elements to be shared, and will govern the sharing of data related to this study. Data will be de-identified, but a link/code is managed within an electronic research management system and maintained by a study coordinator. Data sharing would be allowed if the research is consistent with the original aims of this study and if they have scientific merit as determined by the Principal Investigator.

Sample Size Determination

Power analysis:

The primary endpoint for the protocol is the within-participant difference in fecal energy (via COD) normalized to the total daily energy intake, based to PEG consumption (i.e., COD (kcal) / PEG (g)).

Approach and assumptions in the power analysis (Primary endpoint, Fecal COD): Source data for this power analysis are from the lab of Dr. Krajmalnik-Brown, including replicate samples from 10 participants having within- and between-participant variances, as well as technical replicates. Her lab has extensive experience with the COD measurement (fecal electron content, see fig 2), and replicate variability with that method is approximately 3.0% (n = 10). These variances are in line with those reported for fecal calcium (see TRI's earlier publications on fecal calcium balance). Our power analysis indicates that a sample size of n = 14 (completers) is needed to observe an effect size of roughly 80 kcal/day at 80% power. As our model predicts delta of approximately 110 kcal/day in fecal COD between the two dietary interventions (Fig. 2), we will be sufficiently powered with n = 18 to observe the predicted difference in fecal energy in this study. Importantly, after the first 5 participants have completed, we will measure COD and PEG for the control diet and assess these assumptions and change the sample size, if needed. From reviews of published reports, using PEG administration to normalize fecal energy measurements will decrease variability from 18% to 3%. Thus, we anticipate more power than illustrated by this power analysis. Six consecutive calorimeter stays and measuring a composite of six days of feces and urine will further increase our power.

Other endpoints:
In addition to the bioenergetics of nutrient absorption (the primary endpoint, described above), we will test hypotheses about how changes in the gut microbiota might change enteroendocrine hormone secretion, hunger/satiety, and food intake. Our earlier food intake studies show we are well powered to measure changes in food intake on the order of 200 kcal/day.

Energy expenditure:
For energy expenditure, we will use data from the aforementioned clinical research unit (CRU) study called KEE (NCT01967563), which serves as the basis for many of the procedures employed in this study: namely, strict control of diet energy content (metabolic kitchen), the environment (CRU), and prescribed / observed physical activity. Based on these data on test-retest stability using analogous clinical procedures, we are well powered to detect changes in total daily energy expenditure - and sleeping EE - down to < 6% (approximately 120 kcal/day). We assume no meaningful changes in body composition over 6 days. Lastly, these power analyses were constructed based on two (paired) days in the calorimeter. Because we need 6 days to maximize the power for the primary endpoint our power for ΔEE will be higher. Six consecutive calorimeter stays and measuring a composite of six days of feces and urine will further increase our power.

Statistical Analysis Plan

Aim 1 Statistical approach

We will use repeated measures, and a within-participant model (SAS PROC MIXED with diet and participant as factors in the model) to compare the integrated six-day energy balance for the control vs. ME diets.

Secondary analyses / endpoints, using the same statistical analysis approach, include energy / nutrient absorption (as a % of the ingested calories) for the control vs. ME diets. Once the mechanistic *in silico* model is well developed, we will compare model outputs (predictions) to directly measured (observed) energy absorption using our state-of-the-art metabolic-ward techniques.

Aim 2 Statistical approach
The primary analysis will be the AUC24hour of enteroendocrine secretions. Each hormone will be analyzed independently. Once again, we will use SAS PROC MIXED with diet and participant as factors in the model. Secondary analyses will include diet effects on gastric emptying, bowel transit time, and measured food intake / VAS.

Aim 3 Statistical approach
Microbial metabolism (VCH4 and COD) and human energy metabolism (VO2) will be tested; again, we will use SAS PROC MIXED with diet and participant as factors in the model. Thus, we will be able to quantify the microbial contribution to energy balance and, most importantly, evaluate the feasibility of changing the microbial contribution to energy balance by changing the diet.

Potential Risks and Benefits

Potential Benefits
Participants will likely receive no direct benefit from taking part in this research study. However; research participants may benefit from exercise and/or weight loss and/or behavioral modification.

Potential Risks
Intravenous lines/blood draws (lab samples, e.g.) – The placement of intravenous needles may cause transient pain, vasovagal syncope, and may also result in bruising, bleeding, and/or clotting at the site of needle insertion. The application of direct pressure at the catheterization site will be used to help prevent these symptoms. There is a possibility that a catheter placement would be unsuccessful or need to be removed. If this should occur, another catheter would be placed. It is possible that this may occur more than once during the participant's participation in the protocol. Staff trained and certified in the SOP will be used.

The blood draw volume for this study is higher than the Office for Human Research Protections limit for establishing minimal risk (550 ml in an 8 week period for adults). We are collecting ~700 ml over ~90 days, with the bulk of the blood being collected 4-6 weeks apart: ~346 ml in each study period.

Protection against Risk:
- All venipuncture will be conducted by qualified staff using aseptic techniques to reduce these risks.
- We are recruiting young, healthy individuals and do not anticipate the blood volume for this study to be problematic. However, the following precautions will be taken:

o

- Hemoglobin will be rechecked 3 weeks after Period A and if the levels are <12 g/dl in men, <11 g/dl in women, the PI will review baseline hemoglobin and participants clinical profile to determine the appropriate time for Day 59 enteroendocrine testing.

DEXA scan – there is a very small risk of cancer with excessive exposure to any radiation. Each scan takes about 15 minutes and the radiation dose for five scans is approximately 3 mrem, which is equal to about 3.5 days of background radiation. This radiation exposure is below the guideline of 5000 mrem per year allowed for research participants by the NIH Radiation Safety Committee. The use of the DEXA scan apparatus may cause some minimal discomfort in claustrophobic participants and may cause some minimal back pain in a small minority of the individuals.

Protection against Risk:

- The radiation dose from the scans is less than a chest x-ray, or about the same amount a person would receive from 3.5 days of background radiation from the sun.
- A urine pregnancy test will be done prior to scans of all women of childbearing potential (all women except those with prior hysterectomy, tubal ligation, or absence of menses for ≥2 years).

Hunger and satiety assessment -- There are no known risks associated with hunger and satiety assessment. However, participants will be closely monitored in the event of unexpected choking or other physical distress to the participant. Through this level of monitoring, research staff can intervene immediately.

Radiotransmitter Pill: In adults with no history of previous gastrointestinal surgery, there is a trivially small risk of intestinal blockage involved with the ingestion of this radiotransmitter pill. Adults with a history of gastrointestinal surgery or other gastrointestinal disorders have a greater risk of intestinal blockage. Difficulty swallowing the pill may also be a discomfort for those persons who have trouble swallowing a vitamin-sized pill.

Protection against Risk:

- We will exclude participants from the study if they have a history of gastrointestinal disorders for both safety with regards to this procedure and because these conditions impact the microbiome.
- We will monitor participants when they swallow the pill to monitor for signs of discomfort. Participants who are not able to swallow the pill will not be required to ingest it.

Magnetic Resonance Spectroscopy – There are no known biological risks associated with magnetic resonance spectroscopy. Some short-term discomfort may be experienced. The short-term risks associated with MRI are minimal, but include heating, loud noises and claustrophobia. There are some people who should not undergo MRI; the contraindication is largely based on the presence of certain metal objects within a person (i.e. pacemaker, aneurysm clip, metal fragments, etc.).

Protection against Risk:

- There will be a strict safety screening protocol, to ensure any people with contraindications are excluded from volunteering in MR portion of study
- Incidental Findings: There will be no diagnostic analysis associated with any of the MR sequences used in this protocol; the images will not be sent for radiology review. However some of the MR images we obtain as part of this protocol may show incidental medical findings. In the case where a medical abnormality is apparent on an image, the image will first be reviewed by Steven R. Smith M.D. (the co-investigator on this protocol). If the abnormality is confirmed, then Dr. Smith will recommend that the volunteer seek medical attention from their health care provider.

Heart Rate Monitoring – There are no risks associated with the wearing of monitors, however the chest band that holds the monitor in place may be irritating to the skin for some participants. Participants with nickel allergies may have irritation at the site of the monitor

Protection against Risk:

- Participants with nickel allergies will be excluded from the study.

Activity Monitoring – There are no risks associated with the wearing of activity monitors. However, the armband that holds the BodyMedia monitor in place may be irritating to the skin for some participants. Participants with nickel allergies may have irritation at the site of the monitor

Protection Against Risk:

- Participants with allergies to metal (especially nickel) will not be required to wear the BodyMedia armband.

Inpatient Stay – There are no physical risks associated with the inpatient stay, however some participants may experience feelings such as restlessness, irritability and loneliness.

Protection against Risk:

- Study staff is available at all times for participants to discuss these potential feelings.

Metabolic chamber -- Besides inconveniences that can reasonably be expected as a result of spending an extensive time (24h) in the live-in room calorimeter, there is no risk to participants' physical health. Claustrophobia is an exclusionary criterion. All participants will be given an opportunity to experience the metabolic chamber prior to enrollment in the study.

Protection against Risk:

- Participants will be closely monitored to ensure they are comfortable while in the calorimeter.

Acetaminophen administration -- Allergy and Hypersensitivity - Allergic reactions (primarily rash, pruritic rash, and urticaria) or reports of hypersensitivity (including anaphylaxis) associated

with acetaminophen are very rare and generally are controlled by discontinuation of the drug and, when necessary, symptomatic treatment.

Protection against Risk:

- Participants will be closely monitored to evaluate any reaction to the acetaminophen.

Change in diet -- it is possible that participants will experience GI symptoms on the ME diet such as flatulence, cramping, diarrhea, or constipation.

Protection against Risk:

- Participants will be closely monitored to evaluate GI symptoms.

PEG administration – Precise nutrient balance studies that seek to measure fecal energy and calculate diet energy extraction / digestion require careful consideration of gut transit time and fecal collection.

Traditional methods include the use of non-digestible fecal markers such as carmine red, charcoal, etc. A dose of the marker is given a t = 0 and t = Day 3-7. Fecal material collected before the first appearance of the marker is discarded and all fecal material is collected until the second marker appears. This method is subject to substantial error due to mixing of the intestinal contents during transit and inevitable uncertainties produced during sample collection i.e. determination of pre vs. post marker fecal material.

A second method, continuous marking, uses ingestion of a constant amount of a non-absorbable substance such as barium sulphate, cuprous thiocyanate, chromium sesquioxide or polyethylene glycol [PEG]. Any analyte in the fecal material can then be measured and normalized to the PEG measurement to assess total production *per day as opposed to per gram* - in this case fecal energy and metabolites. We have chosen PEG because of (i) the readily available material (available over the counter in pharmacies), (ii) a precise and accurate method for measuring PEG, (iii) important shortcomings of the other listed substances, (iv) excellent tolerability, and (v) an internal analysis of published literature demonstrates decreased variability when compared to fecal marker methods. Pak concludes: "*This ... method would require a metabolic study as short as 6-7 days ...rather than the customary 12-16 days*" (Pak, 1980). Given that several days are needed to stabilize fecal PEG content (Allen,1979) we will administer PEG for 14 days prior to the collection period and collect samples over 6 days while participants are on the metabolic ward.

PEG will be given at a dose of 0.5g (in a gelatin capsule) TID with meals. Recoveries are reported in the literature at 100% ± 3% (Pak, 1980). We propose a mass-spec measurement based approach which is more accurate and leads to a more precise measurement of PEG.

> **Note on safety and tolerability of PEG:** PEG is used as a laxative and bowel prep at doses ~ 100 times greater than the dose we will use for this protocol. We have now completed a multi-center pilot study (NCT01967563) and we have had ***no adverse events***

or issues with tolerability. This is consistent with its use as a fecal marker in earlier studies from Charlie Pak's lab at UT Southwestern and the safety and tolerability of PEG in medical / clinical settings.

Mitigation of Risks

Invasive procedures (blood sampling and vessel cannulation) will be conducted at the TRI by qualified staff following institutional policies and procedures including sterile dressing to the site.

The most comprehensive and effective method of monitoring risks is a weekly and biweekly individual case that will be reviewed by the PI, the study physician, and/or research coordinator including progress or adverse events occurring in the following: participant confidentiality, participant recruitment, and consent process. The study coordinator is under specific instructions to make the primary investigator aware of all adverse events, expected or unexpected. The following will be monitored: 1) Wound healing after invasive procedures i.e., the IV line placement. The research coordinator will monitor the following items: the timeframe of recruiting participants, quality of data being entered, any external factors relevant for the safety of participants throughout the entire study, and is also instructed to make the PI aware of all events, expected or unexpected. Laboratory tests are obtained during screening phase of study and any abnormal findings are sufficient to exclude individuals. If an abnormal lab is obtained the research participant will be recommended referral to their PCP for follow-up. If illness or injury occurs during a study procedure, participants will be transported to the Emergency Room if needed.

Provisions to Protect the Privacy Interest of Participants

Participants will be assigned unique identifiers for study-related records. All precautions will be taken to make sure that only authorized individuals will access participant research records. The collection of sensitive information about participants will be limited to minimum necessary to achieve the aims of the research, so that no unneeded sensitive information will be collected.

Early Withdrawal of Participants

Investigator Withdrawal of Participants

The participation in this study may be stopped at any time by the study PI without the participant's consent because:

- The study Medical investigator thinks it necessary for participant's health or safety;
- Participant has not followed study instructions;
- Participant is not adherent to the intervention
- The TRI-MD has stopped the study; or
- Administrative reasons require the participant's withdrawal.

Participant Request for Withdrawal from Study

Participation in this study is voluntary. Participants may decide not to participate in this study or may withdraw from this study at any time without penalty or loss of benefits. If a participant leaves the study before the final regularly scheduled visit, s/he may be asked by the study doctor

to make a final visit for some ‘end-of–study’ procedures. This is to make sure that there are no safety concerns.

Data Collection and Follow-up for Withdrawn Participants
Participants who request withdrawal or who are withdrawn by the PI from the study will have their data maintained in the research database up to the point of withdrawal. This data will be included in subsequent analysis because keeping these participants in the analysis is essential for study validity.

Adverse Event Reporting
Adverse Events

An adverse event (AE) is defined as both an expected side effect that is of a serious nature, or an unexpected side effect/event regardless of severity. Each participant is evaluated for adverse events at every study visit. Any event that is reported to the study staff and which meets the criteria of an adverse event will be documented as such and graded as to its attribution (unrelated to protocol, or possibly, probably, or definitely related to protocol) and severity (mild, moderate, or severe). Any severe and/or unanticipated adverse event will be immediately reported to the IRB according to ADVENTHEALTH ORLANDO IRB guidelines.

Recording and Notification of Adverse Events
At each contact with the participant, the investigator will seek information on adverse events by specific questioning and, as appropriate, by examination. Information on all adverse events will be recorded immediately in the source document, and also in the appropriate adverse event module of the case report form (CRF). All clearly related signs, symptoms, and abnormal diagnostic procedures results will be recorded in the source document.

All adverse events will be reported according to AdventHealth Orlando IRB guidelines.

Safety Monitoring Plan

Safety Monitoring
Adverse events will be documented and reported by the study coordinator, Dr. Smith and other TRI staff. Research and safety data will be reviewed by the PI. This review will take place at regular meetings with the research coordinator and study physician where the safety labs for each new participant will be reviewed. Other items discussed will include: progress or adverse events occurring in the following: participant confidentiality, participant recruitment, and consent process. All adverse events and unexpected and/or severe adverse events will be reported to the AdventHealth Orlando IRB. The Translational Research Institute has a standing committee that meets monthly to review all adverse events in our clinical trials and will additionally be charged with review of the study.

Data and Safety Monitoring Board (DSMB) or Equivalent
Given that there is no intervention and does not involve a drug or nutrient we elected not to have a DSM*B*, rather a DSM*P*, instead.

Data and Safety Monitoring Plan (DSMP)

The PI will provide a summary of the data safety and monitoring (DSM) report to NIH on an annual basis, as part of the progress report. The DSM report will include the volunteers, socio-demographic characteristics, expected versus actual recruitment rates, retention rates, any quality assurance or regulatory issues that occurred during the past year, summaries of AEs and SAEs, and any actions or changes with respect to the protocol.

Ethical Considerations
Participation in this study is voluntary. Participants may decide not to participate in this study or may withdraw from this study at any time without penalty or loss of benefits. No vulnerable populations will be studied in this protocol.

Sharing of Results with Participants
Participants will be offered the opportunity to meet with the Principal Investigator or designated medical staff to review the results of their lab assessments or other standard clinical data. Copies of their testing results will be made available to the participants upon request. In addition, the Principal Investigator or designated study staff will provide an overview of the study's outcome to the participant if he or she requests the information.

Conflict of Interest
None of the investigators have any relevant conflicts of interest.

Funding Source
This Study is externally funded through the National Institutes of Health.

Participant Stipends or Payments
For enrolled participants, the first payment will be requested upon completion of Day 10 in the amount of $[redacted] The second payment will be requested upon completion of Day 61 in the amount of up to $[redacted]. A Mastercard® payment will be processed with the dollar amount per visits completed. Mastercard® payments may take up to 3 business days to be processed, once requested. In the event the participant is unable to complete all study visits the payment will be prorated.

Participants who agree to take part in this study will be paid for completed periods according to the following schedule:

Study Periods:
Screening- $[redacted]
Day 1 through Day 10- $[redacted]
Day 11 through Day 20- $[redacted]
Day 21 through Day 61- $[redacted]
TOTAL: $[redacted]

Publication Plan

We attest that the TRI faculty and staff will adhere to POLICY-TRI-ADM-005 (Access to Clinical Trial Data for Publication Purposes).

References

Please refer to grant for a complete list of references.
