# Supplementary material for: Host-diet-gut microbiome interactions influence human energy balance: a randomized clinical trial
Source: Nat Commun. 2023 May 31;14:3161. doi: 10.1038/s41467-023-38778-x (PMC10232526; doi:10.1038/s41467-023-38778-x)
Supplement: Supplementary file 8 — Reporting Summary [file 41467_2023_38778_MOESM8_ESM.pdf]

nature portfolio

Corresponding author(s): Steven R. Smith, Rosa Krajmanik-Brown

Last updated by author(s): May 8, 2023

Reporting Summary

Nature Portfolio wishes to improve the reproducibility of the work that we publish. This form provides structure for consistency and transparency in reporting. For further information on Nature Portfolio policies, see our Editorial Policies and the Editorial Policy Checklist.

Statistics

For all statistical analyses, confirm that the following items are present in the figure legend, table legend, main text, or Methods section.

n/a	Confirmed	
☐	☒	The exact sample size (*n*) for each experimental group/condition, given as a discrete number and unit of measurement
☐	☒	A statement on whether measurements were taken from distinct samples or whether the same sample was measured repeatedly
☐	☒	The statistical test(s) used AND whether they are one- or two-sided<br>*Only common tests should be described solely by name; describe more complex techniques in the Methods section.*
☐	☒	A description of all covariates tested
☐	☒	A description of any assumptions or corrections, such as tests of normality and adjustment for multiple comparisons
☐	☒	A full description of the statistical parameters including central tendency (e.g. means) or other basic estimates (e.g. regression coefficient) AND variation (e.g. standard deviation) or associated estimates of uncertainty (e.g. confidence intervals)
☐	☒	For null hypothesis testing, the test statistic (e.g. *F*, *t*, *r*) with confidence intervals, effect sizes, degrees of freedom and *P* value noted<br>*Give P values as exact values whenever suitable.*
☒	☐	For Bayesian analysis, information on the choice of priors and Markov chain Monte Carlo settings
☒	☐	For hierarchical and complex designs, identification of the appropriate level for tests and full reporting of outcomes
☐	☒	Estimates of effect sizes (e.g. Cohen's *d*, Pearson's *r*), indicating how they were calculated

Our web collection on statistics for biologists contains articles on many of the points above.

Software and code

Policy information about availability of computer code

Data collection	No commercial, open source or custom code was used for data collection.
Data analysis	Statistical analyses were performed using SAS 9.4 and R 4.2.2.<br><br>The other analytical packages utilized for microbiome analysis and visualization are below with versions included in the manuscript: ComplexHeatmap 2.14.0; FastQC 0.12.0; GGplot2 3.4.1; HG28 GRCh38.p14; MaAsLin2 1.12.0; MetaPhlAn3 3.0.14; Microbiome 1.20.0; Phyloseq 1.42.0; R 4.2.2; SAS 9.4; TrimGalore 0.6.5; Vegan 2.6-4.<br><br>The SAS code for the mixed model used to analyze all clinical data and the mathematical model used to in this publication can be found here: https://zenodo.org/badge/latestdoi/634925145

For manuscripts utilizing custom algorithms or software that are central to the research but not yet described in published literature, software must be made available to editors and reviewers. We strongly encourage code deposition in a community repository (e.g. GitHub). See the Nature Portfolio guidelines for submitting code & software for further information.

Data

Policy information about availability of data

All manuscripts must include a data availability statement. This statement should provide the following information, where applicable:

- Accession codes, unique identifiers, or web links for publicly available datasets
- A description of any restrictions on data availability
- For clinical datasets or third party data, please ensure that the statement adheres to our policy

Data availability. Source data are provided with this paper. The raw and processed metagenomic sequence data generated in this study have been deposited in the BioProject database under accession code PRJNA913183 [https://www.ncbi.nlm.nih.gov/bioproject/ PRJNA913183] PRJNA947193 [https://www.ncbi.nlm.nih.gov/ bioproject/PRJNA947193]. The following analytical tools (with respective version numbers) were used in this manuscript: ComplexHeatmap 2.14.0; FastQC 0.12.0; GGplot2 3.4.1; HG28 GRCh38.p14; MaAsLin2 1.12.0; MetaPhlAn3 3.0.14; Microbiome 1.20.0; Phyloseq 1.42.0; R 4.2.2; SAS 9.4; TrimGalore 0.6.5; Vegan 2.6-4.

Human research participants

Policy information about studies involving human research participants and Sex and Gender in Research.

Reporting on sex and gender	We collected information on self-reported sex and reported it in the baseline characteristics table. We did not need to conduct analyses separately by sex due to the crossover design allowing us to evaluate within participant changes on the control diet (Western Diet) and the intervention diet (Microbiome Enhancer Diet). The source data on demographics, including sex, are provided in Source Data files.
Population characteristics	We published the full clinical trial design where details can be obtained on the population and methods: PMID: 32875141; We also provided the study protocol as supplementary material and provided a table with characteristics (Table 1). Since the design was a crossover- there were no relevant co-variates in the analysis.<br><br>Briefly, young, healthy, weight-stable individuals were enrolled to quantify whole body bioenergetics without the confounding effects of age and metabolic disease14, and to establish the comparative data needed for future studies enrolling people with various health conditions. The study sample was 30.8 ± 1.9 years of age, with a BMI within the normal weight to overweight range. All participants reported normal stool patterns based on the Bristol Stool Scale. We excluded people with recent antibiotic use or chronic health conditions by medical history and standard clinical labs.
Recruitment	Participants were recruited from the Orlando, Florida, USA metro area through the use of our database, medical records review and advertising campaigns. Despite selecting a random sample from the general population, with the exception that we intentionally balanced the population by sex, small and time-intensive studies like this one can lead to selection bias, which might limit generalizability. In our case, because each participant serves as their own control and the the treatment sequence allocation was balanced, selection bias is only likely to impact the generalizability to other populations.
Ethics oversight	The study was approved by the AdventHealth Institutional Review board.

Note that full information on the approval of the study protocol must also be provided in the manuscript.

Field-specific reporting

Please select the one below that is the best fit for your research. If you are not sure, read the appropriate sections before making your selection.

- [x] Life sciences
- [ ] Behavioural & social sciences
- [ ] Ecological, evolutionary & environmental sciences

For a reference copy of the document with all sections, see nature.com/documents/nr-reporting-summary-flat.pdf

Life sciences study design

All studies must disclose on these points even when the disclosure is negative.

Sample size	Approach and assumptions in the power analysis (Primary endpoint, Fecal COD): Source data for this power analysis came from replicate samples from 10 participants having within- and between-participant variances, as well as technical replicates. Replicate variability of the COD method was approximately 3.0%. These variances were in line with those reported for fecal calcium. Our power analysis indicates that a sample size of n = 14 (completers) is needed to observe an effect size of roughly 80 kcal/day at 80% power. Our model predicted a delta of approximately 110 kcal/day in fecal COD between the two dietary interventions, thus we were sufficiently powered with n = 18 to observe the predicted difference in fecal energy in this study. From reviews of published reports, using PEG administration to normalize fecal energy measurements will decrease variability from 18% to 3%. Six consecutive calorimeter stays and measuring a composite of six days of feces and urine further increased our power. Thus, we anticipated more power than illustrated by our initial power analysis. For energy expenditure, we used data from a previous study at our facility (NCT01967563) as the basis for many of the procedures employed in this study: strict control of diet energy content (metabolic kitchen), the environment (CRU), and prescribed/observed physical activity. Based on test-retest stability using analogous clinical procedures, we were well powered to detect changes in total daily energy expenditure - and sleeping EE - down to <6% (approximately 120 kcal/day). We assumed no meaningful changes in body composition

	over 6 days. Lastly, these power analyses were constructed based on two (paired) days in the calorimeter. Six consecutive calorimeter days and measuring a composite of six days of feces and urine further increased our power. Indeed, upon completion of all calorimetry measurements on 17 completers, we determined that 26.5 kcal/day is the minimum detectable difference we can expect to at 80% power with 6 repeated measures. Therefore, the study was closed with 17 participants rather than the 18 that were in the original power estimate.<br><br>Citation: PMID: 32875141.
Data exclusions	No data were excluded from the analysis. Due to the crossover design, for each endpoint we analyzed only participants where complete data from both interventions were available. A small number values were considered to be missing at random for the enteroendocrine hormone data (i.e., due to temporary issues with blood draw or laboratory analysis, but not because the entire sample was missing). These values were imputed by the interpolation method (i.e., averaging the previous and subsequent values) for the enteroendocrine hormones and by carrying the last observation forward for the gastric emptying test. Details of these exclusions are within the manuscript.
Replication	The primary drivers of rigor and reproducibility in our study were the immaculate environmental controls within our study design coupled with repeated measures of the primary endpoints and the precision of our methodologies. The primary endpoint data was collected over the 6-day calorimetry period and the total measurements depended on the total number of fecal samples produced (ranging from 4-11 fecal samples/6days). For the primary endpoint- these samples were pooled and this increased power. Our calorimetry measurements were also over a 6-day period (n = 6 measurements per participant) and we show that this replication was successful given the achievement of energy balance depicted in Supplementary Fig 3a. Our approach is completely novel and fills many of the gaps in prior human studies. The fact that all endpoints were collected during the inpatient phase provides exceptional internal validity; should someone else reproduce the study in exactly the same way, we would expect they would achieve the same results.
Randomization	This was a randomized crossover study with a control Western Diet (WD) compared to a Microbiome Enhancer Diet (MBD) where each participant served as their own control, thereby minimizing the impact of confounders68. We applied block randomization stratified by sex. The randomization code was generated by the study statistician who worked directly with the study dietitian in charge of assigning menus to participants. Participants were enrolled by the study coordinator. In order to balance sex, we randomly assigned 3 blocks to each sex. Within each block (n=6), participants were randomly assigned using simple randomization to one of two diet sequences with a 1:1 allocation ratio using SAS PROC PLAN. Eight participants were randomized to sequence 1 (WD followed by MBD) and 9 participants were randomized to sequence 2 (MBD followed by WD).<br><br>Citation: PMID: 32875141
Blinding	Although it was not possible to blind the participants or front-line staff to the components of the diet, as the food items could reveal which diet was being dispensed, the investigators were, as much as possible, not present when meals were dispensed. Importantly, the diet assignment was NOT tied to any data streams throughout the entire course of the study. During the analysis phase, data streams remained blinded for all individuals involved in the study (including the investigators) until results were locked by the biostatistician who held the randomization code. Any analyses performed by other individuals of the study team were performed blinded to diet assignment. Once results were locked by the biostatistician, the statistical analysis was unblinded.<br><br>Citation: PMID: 32875141

Reporting for specific materials, systems and methods

We require information from authors about some types of materials, experimental systems and methods used in many studies. Here, indicate whether each material, system or method listed is relevant to your study. If you are not sure if a list item applies to your research, read the appropriate section before selecting a response.

Materials & experimental systems

n/a	Involved in the study	
☒	☐	Antibodies
☒	☐	Eukaryotic cell lines
☒	☐	Palaeontology and archaeology
☒	☐	Animals and other organisms
☐	☒	Clinical data
☒	☐	Dual use research of concern

Methods

n/a	Involved in the study	
☒	☐	ChIP-seq
☒	☐	Flow cytometry
☒	☐	MRI-based neuroimaging

Clinical data

Policy information about clinical studies
All manuscripts should comply with the ICMJEguidelines for publication of clinical research and a completedCONSORT checklist must be included with all submissions.

Clinical trial registration	NCT02939703
Study protocol	The full protocol is included as Supplementary Dataset 2. In addition, we published the study design here: PMID: 32875141
Data collection	All clinical samples and data were collected at at the AdventHealth Translational Research Institute in Orlando, Florida, USA. Participants were enrolled between June of 2017 and August of 2019. The last participant visit occurred in October of 2019 and this completed the data collection phase.
Outcomes	The primary endpoint for the protocol was the within-participant difference in 24-h fecal energy, as measured by COD, normalized to the total daily energy intake and to the non-metabolizable marker PEG [COD (mg/g stool)/PEG (g)]. We were testing the hypothesis that fecal COD will be higher on the Microbiome Enhancer Diet vs. the Western Diet.<br><br>The principal secondary endpoints tested hypotheses about how changes in the gut microbiota might change enteroendocrine hormone secretion, hunger/satiety, and food intake.<br><br>Citation: PMID: 32875141
